# Virology-aware artificial intelligence for respiratory antiviral discovery: from molecular docking biases to RdRp-centered benchmarking

**DOI:** 10.3389/fphar.2026.1940098

**Published:** 2026-10-15

**Authors:** Luis Ruben Ramos-Cortes, Alan Orlando Santos-Mena, Luis Enrique Flores-Padilla, Kevin Paul Miranda-Lizárraga, David Mauricio Cañedo-Figueroa, Luisa Fernanda Ontiveros-Zúñiga, Flor Itzel Lira-Hernández, Juan Fidel Osuna-Ramos, Saúl Noriega, Jaime Cardoso-Ortiz, Vanessa Alcalá-Ramírez, Luis Adrián De Jesús-González

**Affiliations:** 1 Laboratorio de Virología Molecular, Unidad de Investigación Biomédica de Zacatecas, Instituto Mexicano Del Seguro Social, Zacatecas, Mexico; 2 Unidad Académica de Ingeniería Eléctrica, Universidad Autónoma de Zacatecas, Zacatecas, Mexico; 3 Unidad Académica de Ciencias Químicas, Universidad Autónoma de Zacatecas, Zacatecas, Mexico; 4 Laboratorio de Virología y Diseño de Antivirales, Facultad de Medicina, Universidad Autónoma de Sinaloa (UAS), Culiacán, Mexico; 5 Laboratorio de Biotecnología II, Facultad de Ciencias Químicas, Universidad Autónoma de Chihuahua, Chihuahua, Mexico

**Keywords:** antiviral discovery, artificial intelligence, benchmarking, generative AI, machine learning, molecular docking, protein-ligand affinity, respiratory RNA viruses

## Abstract

Respiratory RNA viruses remain a persistent threat because they combine high transmissibility, rapid evolution, immune escape, zoonotic spillover, and incomplete therapeutic coverage. RNA-dependent RNA polymerases (RdRps) and polymerase complexes are among the most conserved and pharmacologically actionable viral targets, yet they are also challenging computational targets because their ligandable states depend on RNA templates, cofactors, divalent metals, multidomain architecture, and conformational transitions. Artificial intelligence (AI), machine learning (ML), deep learning (DL), geometric deep learning (GDL), and generative molecular design are increasingly used to accelerate antiviral discovery, yet their performance is often inferred from general protein-ligand benchmarks dominated by non-viral targets. This review argues that respiratory RNA virus polymerases are a high-value but comparatively underrepresented class of antiviral targets and a complementary test case for evaluating AI-guided drug discovery. We synthesize current approaches in computer-aided drug design (CADD), molecular docking, ML-assisted virtual screening, molecular representations, pose-generation models, generative AI, and protein-ligand affinity benchmarking. We emphasize that docking scores, predicted affinity, and root-mean-square error (RMSE) values are insufficient unless linked to target engagement, enzymatic inhibition, cell-based antiviral activity, cytotoxicity, resistance, and pharmacological feasibility. We propose an RdRp-centered AI pipeline that integrates curated polymerase structural ensembles, leakage-resistant benchmarking, antiviral-specific evaluation tasks, ADME/Tox filtering, biochemical validation, cell-based infection assays, resistance profiling, and iterative model refinement. Moving beyond docking scores requires benchmarking models under virologically realistic conditions and validating whether computational prioritization enriches compounds with experimentally actionable antiviral activity.

## 1 Introduction

Respiratory RNA viruses remain a persistent threat to global health because they combine high transmissibility, recurrent emergence, antigenic variability, zoonotic potential, and limited availability of broad-spectrum therapeutics. Severe acute respiratory syndrome coronavirus 2 (SARS-CoV-2), influenza viruses, respiratory syncytial virus (RSV), human metapneumovirus (hMPV), and human parainfluenza viruses (HPIVs) illustrate a broader pharmacological challenge: vaccines and monoclonal antibodies have reshaped prevention for selected pathogens, but small-molecule antivirals remain essential for treating infected individuals, protecting high-risk populations, responding to immune escape, and controlling outbreaks caused by novel or re-emerging variants. The rapid pace of RNA virus evolution further complicates antiviral development because therapeutic candidates must not only inhibit viral replication but ideally retain activity across genetically diverse strains, related viral species, and resistance-prone replication contexts (Sadybekov and Katritch, 2023; Tirosyan et al., 2026).

Artificial intelligence (AI) has entered antiviral drug discovery in response to this urgency. Recent AI-guided approaches have been applied to antiviral target identification, prediction of host-virus protein-protein interactions, machine learning (ML)-assisted interpretation of genome-wide CRISPR screens, drug repurposing, *de novo* molecule generation, and prediction of resistance-associated mutations from viral sequence data. In principle, these methods can accelerate multiple stages of antiviral discovery by identifying candidate targets, prioritizing compounds, exploring chemical space, predicting protein-ligand interactions, optimizing multiparameter drug-like profiles, and integrating heterogeneous biological datasets (Patel et al., 2020; Fleming and Ivanov, 2026). However, the field remains uneven: although computationally sophisticated studies are increasingly common, current evidence indicates that comparatively few AI-nominated antiviral candidates have undergone comprehensive biochemical, cellular, *in vivo*, or resistance-validation pipelines (Tirosyan et al., 2026).

The COVID-19 pandemic intensified interest in AI-guided antiviral discovery because conventional experimental screening and medicinal chemistry timelines are ill-suited to rapidly evolving outbreaks. AI-guided workflows were used to prioritize existing compounds, generate new chemical entities, and screen ultra-large libraries against viral targets (Beck et al., 2020; Kc et al., 2021). In the Billion Molecules against COVID-19 Challenge, summarized by (Tirosyan et al., 2026), AI-based teams selected 557 synthesized molecules, yielding 22 compounds with measurable biological activity, whereas traditional virtual-screening teams selected 372 synthesized molecules and identified 1 active compound. This reported comparison suggests that AI-guided prioritization may improve enrichment efficiency within well-defined screening settings; however, this result should not be generalized beyond the specific targets, compound libraries, computational strategies, and experimental conditions evaluated. Moreover, improved hit enrichment does not necessarily translate into pharmacological robustness or clinical relevance.

Among respiratory RNA-virus targets, polymerase complexes are especially attractive because they combine essentiality, catalytic conservation, and pharmacological precedent. Their computational treatment is nevertheless challenging because the relevant ligandable state can depend on accessory subunits, RNA, divalent metals, and conformational transitions. These features motivate the RdRp-centered scope of this review and are examined comparatively in Section 2 (Pathania et al., 2022).

Most AI and ML models for protein-ligand affinity prediction have not been developed specifically for viral polymerases. Instead, they are commonly trained and benchmarked on general protein-ligand datasets dominated by non-viral targets, crystallographically resolved complexes, recurrent chemotypes, and relatively well-characterized binding pockets. This creates a transferability problem: high performance on a general benchmark does not necessarily translate into reliable predictions for underrepresented, flexible, cofactor-dependent viral targets (Sadybekov and Katritch, 2023; Tirosyan et al., 2026). The present manuscript therefore argues that antiviral AI should not be evaluated solely by generic metrics such as retrospective affinity correlation, RMSE, area under the curve, or docking success rate. These metrics are informative but insufficient unless linked to the biological and pharmacological requirements of antiviral discovery.

Recent advances in deep learning (DL)-based molecular modeling make this discussion timely. Geometric deep learning (GDL), graph neural networks (GNNs), equivariant graph neural networks (EGNNs), diffusion-based docking models, and neural scoring functions are reshaping structure-based drug discovery by incorporating three-dimensional molecular geometry, symmetry, and learned protein-ligand interaction patterns (Isert et al., 2023; Zhou et al., 2023; Zhang et al., 2025; Isert et al., 2023) emphasized that structure-based drug design increasingly uses three-dimensional representations, such as grids, surfaces, and graphs, to address tasks including molecular property prediction, binding-site identification, pose prediction, and *de novo* design. Similarly, KarmaDock illustrates technical progress in DL-based docking by integrating docking acceleration, binding pose generation and correction, and binding-strength estimation within a single framework (Zhang et al., 2023). Yet these advances expose a recurring issue: models that perform well on curated benchmarks may still struggle when applied prospectively to new target families, altered receptor states, cryptic pockets, induced-fit mechanisms, RNA-bound conformations, or experimentally noisy antiviral endpoints.

Generative AI adds another layer to this transformation. Rather than merely screening existing libraries, generative models can propose new molecular structures, optimize scaffolds, explore analog series, and balance multiple properties simultaneously (Ozdemir et al., 2026). This is especially relevant for antiviral discovery, where nucleoside and non-nucleoside analogs often require fine-tuning of potency, selectivity, activation, permeability, metabolic stability, and resistance profile. However, generative validity is not equivalent to antiviral validity. A molecule can be syntactically valid, chemically plausible, novel, and computationally favored yet still fail due to poor solubility, inadequate cell permeability, lack of intracellular activation, metabolic instability, toxicity, or absence of target engagement in infected cells (Tirosyan et al., 2026).

Numerous recent reviews have outlined the overarching framework of AI in target identification, drug repurposing, *de novo* design, and viral resistance prediction. We focus on a specific, pharmacologically significant question: how can AI-guided docking, affinity prediction, molecular representation learning, benchmarking, and generative design be tailored to RdRp-focused antiviral discovery targeting respiratory RNA viruses? Respiratory RNA virus polymerases constitute a significant target class for the development of broad-spectrum antivirals and provide a complementary, rather than inherently superior, test of the practical applicability of AI-guided drug discovery. Their value as a test case arises from their conservation, structural complexity, conformational plasticity, dependence on RNA, cofactors, and metal ions, limited target-specific datasets, and demanding experimental validation requirements. In contrast, viral proteases, particularly SARS-CoV-2 Mpro, currently have more mature prospective evidence for AI-supported inhibitor discovery, including the Chemistry42-designed inhibitor ISM3312 (Sun et al., 2025). We therefore frame RdRp as a comparatively underexplored target family for testing model transferability under different biological constraints, rather than as a “better” target than viral proteases.

## 2 Respiratory RNA virus polymerases as high-value antiviral targets

Rather than restating the essential role of polymerase, this section examines how family-specific architecture alters the druggable system presented to computational models. For direct-acting antiviral discovery, the relevant question is whether conserved catalytic or allosteric features remain structurally accessible across biologically realistic receptor states. This criterion distinguishes polymerases from targets whose tractability depends predominantly on transient interfaces or highly variable antigenic surfaces (Te Velthuis, 2014; Bonneux et al., 2024).

Polymerase targeting is not universally preferable, and target selection should depend on the biological objective and the practical constraints of each discovery campaign. RdRp-centered strategies are particularly attractive when the desired profile includes catalytic essentiality, conservation across related viruses, the potential for broad-spectrum inhibition, and access to mechanistically interpretable polymerase assays. Proteases may be preferable when well-defined catalytic pockets, abundant structural information, robust biochemical assays, and rapid compound optimization are available. Entry or fusion proteins may be more suitable for prophylactic or early-intervention strategies when extracellular accessibility and blockade of the initial infection step are priorities. Influenza neuraminidase, M2, and the PA endonuclease remain validated alternatives when virus-specific intervention is appropriate, whereas host-directed targets may offer broader coverage and a potentially higher resistance barrier but introduce additional concerns regarding toxicity, redundancy, and cell-context dependence. Target selection should therefore consider conservation, structural druggability, assay availability, therapeutic timing, resistance liability, safety, and the intended viral spectrum. The RdRp-centered focus of this review should be understood as a deliberate analytical framework rather than a claim that polymerases are universally superior to other antiviral targets (Jo et al., 2022; Malone et al., 2022; Von Delft et al., 2023; Bonneux et al., 2024; Bonomini et al., 2025).

Respiratory RNA viruses encode polymerase systems that differ in architecture but converge in function. In coronaviruses, including SARS-CoV-2, the catalytic polymerase is nsp12, which functions with cofactors such as nsp7 and nsp8 within a larger replication-transcription complex. This complex synthesizes genomic and subgenomic viral RNA and is further influenced by accessory enzymes involved in proofreading, capping, and RNA processing (Vicenti et al., 2021; Malone et al., 2022). The clinical and experimental relevance of this target class is underscored by the success of nucleoside analog strategies and the continued interest in non-nucleoside inhibitors that modulate polymerase activity through catalytic, allosteric, or interface-associated mechanisms.

Influenza viruses present a distinct structural paradigm. Their polymerase is a heterotrimeric PA-PB1-PB2 complex associated with viral ribonucleoproteins. PB1 contains the core polymerase activity, PA provides the endonuclease function required for cap-snatching, and PB2 participates in cap recognition and host-adaptive interactions. This division of function creates multiple ligandable opportunities within a single replication apparatus. Approved and investigational influenza polymerase inhibitors demonstrate that viral polymerase complexes can be pharmacologically targeted at distinct mechanistic nodes, including nucleotide incorporation, endonuclease activity, and transcript initiation (Te Velthuis and Fodor, 2016; Bonomini et al., 2025).

For pneumoviruses and paramyxoviruses such as RSV, hMPV, and HPIV, polymerase activity resides in the large L protein, a multifunctional enzyme that integrates RNA synthesis with capping and methyltransferase activities (Bonneux et al., 2024). L requires association with phosphoprotein cofactors and operates on nucleocapsid-associated RNA rather than naked RNA templates. This organization makes the polymerase both attractive and challenging: attractive because L is essential and conserved within related virus groups, but challenging because several functionally relevant conformations and interfaces are difficult to capture in a single static structure. In this context, the biologically relevant target is a dynamic polymerase system, not a rigid protein prepared for a single docking run (Bonneux et al., 2024; Ontiveros-Zúñiga et al., 2026). The principal domain architectures and functional polymerase complexes of SARS-CoV-2, influenza A virus, RSV, hMPV, and HPIV are compared in Figure 1.

**FIGURE 1:**
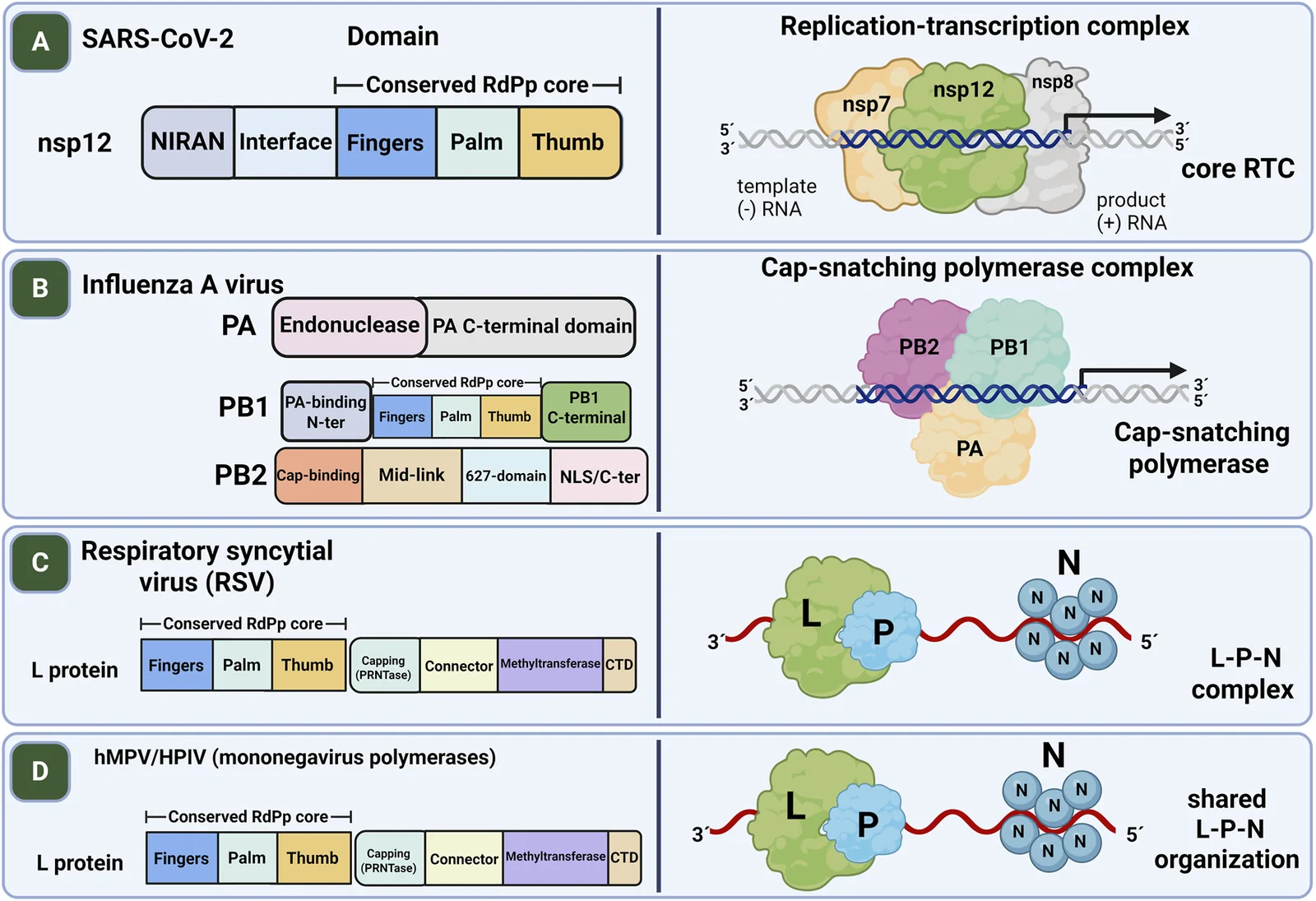
Structural organization of polymerase systems from major respiratory RNA viruses. Schematic comparison of the domain architecture and functional complexes of SARS-CoV-2, influenza A virus, respiratory syncytial virus (RSV), human metapneumovirus (hMPV), and human parainfluenza virus (HPIV). **(A)** SARS-CoV-2 nsp12 contains the NiRAN and interface domains followed by the conserved fingers, palm, and thumb RdRp core. Nsp12 associates with nsp7 and nsp8 to form the core replication–transcription complex and synthesize positive-sense RNA from a negative-sense template. **(B)** The influenza polymerase is a heterotrimeric PA–PB1–PB2 complex: PA contains the cap-snatching endonuclease, PB1 harbors the conserved RdRp core, and PB2 mediates cap binding and regulatory functions. **(C)** The RSV L protein contains RdRp, capping, connector, methyltransferase, and C-terminal domains and functions with the P cofactor on N-encapsidated RNA. **(D)** hMPV and HPIV share the characteristic mononegavirus L–P–N organization. These systems illustrate conserved catalytic features alongside virus-specific cofactors, RNA contexts, accessory domains, and potential allosteric or interfacial targets. The replication complexes are intentionally represented schematically to emphasize their functional organization and the viral components participating in RNA synthesis rather than atomistic structural detail. Created with BioRender. De Jesus Gonzalez, L. A. (2026). https://BioRender.com/9ara6ys.

Across these respiratory RNA viruses, the term “polymerase target” therefore encompasses more than the catalytic palm domain of a single enzyme. It includes catalytic sites, nucleotide-entry channels, RNA-binding grooves, cofactor interfaces, interdomain regulatory pockets, capping domains, and transient conformational states. This broader definition is important because AI-guided docking and affinity prediction may identify ligands that do not act as classical active-site inhibitors. Non-nucleoside compounds can act through allosteric stabilization, conformational trapping, cofactor disruption, or interference with polymerase complex assembly. Polymerase-centered antiviral discovery should therefore not be reduced to docking nucleoside analogs into a conserved active site (Te Velthuis, 2014; Te Velthuis and Fodor, 2016; Malone et al., 2022; Bonneux et al., 2024; Bonomini et al., 2025).

These architectures frame the central modeling problem addressed throughout the remainder of this review: the pharmacologically relevant target is not a single static protein but an ensemble whose ligandable states depend on RNA, cofactors, metals, and domain organization. The next section evaluates how conventional computer-aided drug design (CADD) and docking workflows capture or fail to capture this biological context (Te Velthuis, 2014; Te Velthuis and Fodor, 2016; Malone et al., 2022; Bonneux et al., 2024; Bonomini et al., 2025).

## 3 From computer-aided drug design to conventional docking in antiviral discovery

The focus on respiratory RNA virus polymerases naturally raises the methodological question of how to interrogate these targets early in antiviral discovery. Because RdRp and polymerase complexes combine conserved catalytic functions with substantial conformational complexity, prioritizing them requires approaches that translate structural information into experimentally testable ligand hypotheses. CADD provides this bridge between structural virology, medicinal chemistry, and pharmacology. In its broadest sense, CADD encompasses computational strategies for identifying, prioritizing, optimizing, or designing molecules with potential biological activity against defined targets. In antiviral discovery, CADD has been particularly useful when structural data are available for viral enzymes, entry proteins, capsid components, or replication complexes, enabling researchers to move from target selection to compound prioritization before committing to resource-intensive biochemical or cell-based assays (Murgueitio et al., 2012; Isert et al., 2023).

In classical structure-based workflows, CADD typically begins with a target structure. This structure may come from X-ray crystallography, cryo-electron microscopy, nuclear magnetic resonance, homology modeling, or modern protein structure prediction (Wong et al., 2022; Ren et al., 2023). For viral polymerases, the choice of structure is not a trivial technical step. A SARS-CoV-2 nsp12 structure without cofactors or RNA may highlight different cavities than a complete nsp12-nsp7-nsp8-RNA complex; an influenza polymerase conformation captured during transcription may differ from one associated with replication; and RSV L protein structures may represent only selected conformational states of a large multidomain enzyme. Thus, receptor preparation is already a biological decision: protonation states, missing loops, cofactors, metal ions, RNA fragments, water molecules, and oligomeric context can influence the predicted ligand-binding landscape (Verdonk et al., 2003; Trott and Olson, 2010; Murgueitio et al., 2012; Sterling and Irwin, 2015).

Once a receptor state is selected, VS is commonly used to prioritize compounds from chemical libraries. These libraries may include approved drugs for repurposing, investigational antivirals, natural products, nucleoside analogs, non-nucleoside inhibitor-like scaffolds, focused antiviral libraries, or ultra-large purchasable chemical spaces (Gaulton et al., 2012; Irwin et al., 2020). The objective is not to prove antiviral activity computationally but to reduce a large chemical search space to a smaller set of candidates that merit deeper evaluation. This step is increasingly important because modern chemical libraries can contain hundreds of millions to billions of compounds. KarmaDock positions docking as a core technology for structure-based VS and highlights the pressure created by ultra-large compound libraries such as ZINC20, which contains more than 230 million purchasable compounds (Zhang et al., 2023).

Molecular docking is the central operation in many structure-based VS workflows. Docking aims to predict how a small molecule might bind within a defined region of a macromolecular target and to estimate the relative favorability of that pose. Classical docking programs such as AutoDock, AutoDock Vina, Glide, GOLD, and related tools typically combine a conformational search algorithm with a scoring function. The search algorithm explores possible ligand orientations and conformations, and in some implementations, limited receptor flexibility; the scoring function ranks poses using simplified energetic approximations. These approximations may include steric complementarity, hydrogen bonding, electrostatic interactions, hydrophobic contacts, van der Waals interactions, desolvation terms, and empirical penalties (Yang et al., 2022; Zhang et al., 2023).

For antiviral discovery, docking offers several practical advantages. It can rapidly prioritize compounds, suggest plausible binding modes, identify recurring interactions with conserved residues, compare candidate pockets, guide analog selection, and support mechanistic hypotheses. When applied cautiously, docking can help distinguish molecules that are structurally incompatible with a target from those that merit further analysis. In RdRp-centered discovery, docking may be used to explore nucleotide-binding sites, nascent RNA channels, allosteric pockets, interdomain cavities, cofactor interfaces, or capping-associated domains. It can also support repositioning campaigns by mapping approved drugs or known bioactive compounds onto viral proteins, a strategy widely used during the COVID-19 pandemic (Verdonk et al., 2003; Trott and Olson, 2010; Murgueitio et al., 2012; Sterling and Irwin, 2015; Yang et al., 2022; Von Delft et al., 2023; Zhang et al., 2023).

A typical docking-driven antiviral workflow involves several sequential decisions. First, the biological target is selected based on essentiality, conservation, and druggability. Second, one or more structural states are prepared, with decisions on protonation, cofactors, RNA, water molecules, and binding-site boundaries. Third, a ligand library is curated, standardized, protonated, filtered for physicochemical properties (Bickerton et al., 2012), and converted into three-dimensional conformations. Fourth, docking is performed into a predefined pocket or, less commonly, via blind docking across a broader surface. Fifth, poses are ranked by score, interaction patterns, chemical plausibility, and consistency with known structure-activity relationships. Sixth, top candidates may undergo rescoring, molecular dynamics (MD), binding free-energy estimation, ADME filtering, or comparison across receptor conformations. Finally, experimentally testable compounds are selected for biochemical and cellular validation (Verdonk et al., 2003; Trott and Olson, 2010; Murgueitio et al., 2012; Sterling and Irwin, 2015; Zhang et al., 2023).

Defining pockets in respiratory RNA virus polymerases is one of the most consequential steps. Active sites are often conserved yet crowded, polar, metal-dependent, or nucleotide-like, making them favorable for nucleoside analogs but challenging for chemically diverse non-nucleoside compounds. By contrast, allosteric or interdomain sites may be more chemically permissive but less conserved or more conformation-dependent. In SARS-CoV-2, candidate pockets may be evaluated within the catalytic nsp12 core, in RNA-adjacent channels, or near cofactor-associated regions. In influenza, docking opportunities are distributed across PA, PB1, PB2, and intersubunit interfaces. In RSV and hMPV, the multifunctional L protein introduces possible pockets in polymerase, capping, and methyltransferase-associated domains (Verdonk et al., 2003; Trott and Olson, 2010; Murgueitio et al., 2012; Sterling and Irwin, 2015).

Accordingly, docking scores are treated here as prioritization outputs rather than standalone evidence (Lam and Katritch, 2025). Their metric interpretation is addressed in Section 9.2, and their role within the proposed stage-gated workflow is operationalized in Section 13.

Docking can be strengthened when embedded within broader CADD workflows. Redocking and cross-docking can be used to assess whether a protocol reproduces known ligand poses. Ensemble docking can evaluate multiple receptor conformations and reduce reliance on a single static structure. MD can test whether predicted poses remain stable over time, reveal induced-fit effects, expose transient pockets, and characterize protein-ligand interactions under more realistic dynamics. Free-energy methods such as molecular mechanics/Poisson-Boltzmann surface area (MM/PBSA) or molecular mechanics/generalized Born surface area (MM/GBSA) can provide additional ranking information, although their reliability depends on system preparation, sampling, and parameterization (Tondar et al., 2024). Tirosyan et al. (2026) described experimentally validated generative AI-designed influenza neuraminidase inhibitors whose development incorporated AutoDock Vina docking, synthetic accessibility and drug-likeness scoring, MM/PBSA rescoring, and MD before *in vitro* and *in vivo* validation (Tirosyan et al., 2026).

CADD has played and will continue to play an important role in antiviral discovery, but its primary function is prioritization rather than confirmation. For respiratory RNA virus polymerases, this distinction is critical because the relevant targets are dynamic complexes whose ligandable states may depend on RNA, cofactors, accessory domains, and conformational transitions. Classical docking provides a necessary starting point: it enables structure-guided triage of chemical space and generates molecular hypotheses. However, its limitations justify the next layer of the review. In the literature reviewed here, we did not identify a standardized quantitative relationship between docking scores and biochemical IC_50_ or cellular EC_50_ values for respiratory-virus RdRps across sufficiently large and consistently tested sets of compounds. Accordingly, our argument is primarily precautionary and mechanistic rather than a claim of a universal measured correlation. In one series of target-specific HCV NS5B inhibitors, GOLD fitness scores showed an r^2^ of 0.67 with experimental bioactivity, leaving approximately one-third of the observed variance unexplained (Azam et al., 2014). This single HCV result should not be generalized to respiratory-virus polymerases. Because docking scores estimate compatibility with a modeled binding pose, whereas IC_50_ and EC_50_ values also depend on receptor state, RNA, cofactors, metal ions, assay conditions, permeability, metabolism, and cytotoxicity, RdRp-centered discovery requires complementary methods that can learn from data, handle molecular representations more flexibly, accelerate virtual screening, and integrate structural uncertainty (Verdonk et al., 2003; Trott and Olson, 2010; Murgueitio et al., 2012; Sterling and Irwin, 2015; Zhang et al., 2023). This methodological progression, from conventional structure-based virtual screening to ML-assisted triage, geometric pose generation, and iterative generative design, is summarized in Figure 2.

**FIGURE 2:**
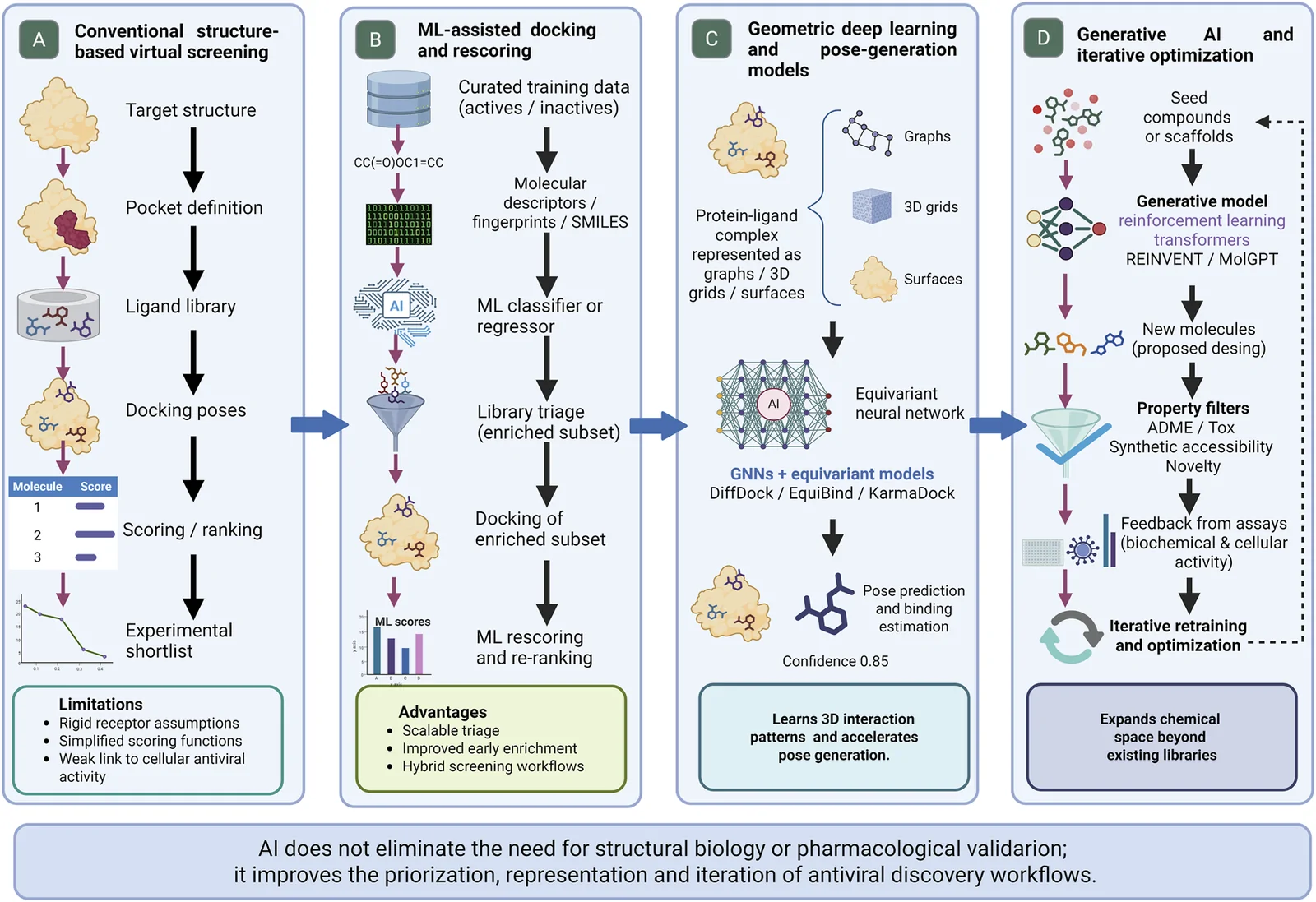
From conventional virtual screening to AI-guided antiviral molecule design. Comparison of four complementary computational strategies. **(A)** Conventional structure-based virtual screening involves target preparation, pocket definition, ligand-library selection, docking, scoring, ranking, and experimental shortlisting, but remains limited by rigid-receptor assumptions, simplified scoring functions, and weak correspondence with cellular antiviral activity. **(B)** Machine learning (ML)-assisted workflows use curated training data and molecular descriptors, fingerprints, or SMILES strings to triage large libraries before docking, rescoring, and reranking an enriched subset. **(C)** Geometric deep-learning models represent protein–ligand complexes as graphs, three-dimensional grids, or molecular surfaces. Equivariant neural networks, including DiffDock, EquiBind, and KarmaDock, learn three-dimensional interaction patterns for pose generation and binding estimation. **(D)** Generative AI uses seed compounds or scaffolds to propose new molecules, which are filtered according to ADME/Tox properties, synthetic accessibility, and novelty. Biochemical and cellular results then support iterative model retraining and molecular optimization. AI therefore complements structural biology and pharmacological validation by improving prioritization, molecular representation, and exploration of chemical space. Created in BioRender. De Jesus Gonzalez, L. A. (2026) https://BioRender.com/oy4h8ry.

## 4 Why conventional docking struggles with dynamic viral polymerases

Conventional molecular docking remains one of the most widely used entry points for structure-based antiviral discovery, but its predictive power diminishes when applied to dynamic viral polymerases. Respiratory RNA virus polymerases are not rigid receptors awaiting ligand accommodation; they are conformationally active molecular machines whose ligandability depends on catalytic state, RNA engagement, nucleotide binding, metal coordination, cofactor assembly, and interdomain communication. Docking can therefore be useful for hypothesis generation but is insufficient as evidence of antiviral mechanism or therapeutic potential (Tanimoto et al., 2022; Yang et al., 2022; Tirosyan et al., 2026).

The primary limitation in applying docking to dynamic viral polymerases lies in target representation rather than scoring alone. Standard docking captures an isolated receptor conformation, whereas polymerase activity is governed by coordinated transitions during initiation, elongation, and cofactor assembly (Tanimoto et al., 2022; Yang et al., 2022; Tirosyan et al., 2026). Consequently, a top-ranked pose represents a static geometric fit that does not account for induced-fit phenomena, dynamic RNA engagement, or physiological target accessibility (see Section 12 for the experimental validation cascade).

This problem is especially relevant to RdRp-directed discovery because the target is central to replication yet not automatically accessible to every compound that docks well. The replication complex may reside in specialized intracellular compartments, associated with viral or host membranes, or be embedded in ribonucleoprotein structures. A ligand must therefore reach the right cell type, enter the cell, avoid degradation or efflux, access the replication compartment, and engage the correct polymerase state at sufficient concentration. Docking captures none of these pharmacological constraints (Tanimoto et al., 2022; Yang et al., 2022; Tirosyan et al., 2026).

Many conventional docking workflows rely on a static receptor conformation. Even when limited side-chain flexibility is allowed, the receptor is often treated as largely fixed. This assumption is convenient for speed but conflicts with the biology of viral polymerases. RdRps and polymerase complexes undergo coordinated conformational transitions during initiation, elongation, translocation, nucleotide incorporation, proofreading-associated events, capping, and termination. Tanimoto et al. (2022) reviewed MD studies of SARS-CoV-2 RdRp and emphasized the dynamic nature of polymerase complexes during nucleotide incorporation and inhibitor recognition (Tanimoto et al., 2022).

For viral polymerases, receptor preparation is a biological decision rather than a purely computational preprocessing step. Polymerase active sites and regulatory pockets are shaped by RNA templates, nascent RNA products, nucleotide substrates, divalent metals, protein cofactors, and sometimes structured water networks. Removing these elements can simplify the docking system but distort the actual binding environment. Conversely, retaining them without a clear rationale can introduce artificial steric constraints, unrealistic electrostatics, or inaccessible binding sites (Tanimoto et al., 2022; Yang et al., 2022; Tirosyan et al., 2026).

Nucleoside and nucleotide analogs reveal why target-only docking is incomplete. For this chemotype, antiviral activity is not determined solely by the polymerase’s recognition of the parent compound. Many analogs require cellular uptake, sequential phosphorylation, conversion to active triphosphate forms, sufficient intracellular accumulation, and competition with endogenous nucleotide pools. A docking study using the wrong chemical form may yield structurally interesting but pharmacologically misleading results.

Docking output is also shaped by the search space, sampling density, ligand conformer generation, protonation state, tautomer selection, grid definition, scoring function, and post-docking filtering. These parameters are often treated as technical details, yet they can determine whether a compound is classified as a hit or discarded. For dynamic targets, insufficient sampling may yield false negatives by failing to explore relevant poses, whereas permissive sampling may yield false positives (Tanimoto et al., 2022; Yang et al., 2022; Tirosyan et al., 2026).

Because a cellular antiviral phenotype does not, by itself, establish engagement of the docked polymerase, mechanistic confirmation should follow the target-engagement and experimental validation cascade, as described in Section 12.

The limitations of conventional docking do not imply that docking should be abandoned. Rather, they define the problem that AI-guided approaches must solve. ML-based scoring, GDL, and pose-generation models can accelerate VS, learn complex interaction patterns, and incorporate richer molecular representations. However, these approaches inherit many of the same vulnerabilities when trained on biased datasets, evaluated on static crystallographic complexes, or assessed solely by retrospective affinity metrics (Tanimoto et al., 2022; Yang et al., 2022; Tirosyan et al., 2026).

## 5 Machine learning as an alternative and complement to docking

The limitations of conventional docking for dynamic viral polymerases underscore the need for scalable, data-aware, and biologically constrained computational workflows (Sim et al., 2025). ML has emerged not as a simple replacement for docking but as a set of methods that can accelerate compound triage, improve scoring, learn complex molecular representations, support hybrid screening strategies, and prioritize candidates before experimental validation (McNutt et al., 2021; Yang et al., 2022; Zhang et al., 2023; Bande and Baday, 2024; Sindt and Rognan, 2026).

### 5.1 Computational efficiency and ultra-large virtual screening

One of the strongest arguments for ML-assisted docking is its computational scalability. Classical VS requires explicit evaluation of large numbers of protein-ligand pairs, often through repeated pose generation and scoring. As compound libraries continue to expand, exhaustive docking of every molecule against every relevant polymerase conformation becomes impractical. This computational bottleneck is particularly relevant for RdRp-centered screening: ML-based scoring and prioritization can serve as scalable filters, reducing the number of molecules that require explicit docking across large libraries and multiple receptor states (Zhang et al., 2023; Sindt and Rognan, 2026).

KarmaDock illustrates how DL is beginning to reshape this efficiency problem. Zhang et al. (2023) describe KarmaDock as a DL-based docking method that integrates docking acceleration, pose generation and correction, and binding-strength estimation. Its architecture includes protein and ligand encoders, E(n)-equivariant GNNs with self-attention, post-processing to preserve chemically plausible structures, and a mixture density network for scoring. In the comparison reported by Zhang et al. (2023), KarmaDock processed the same 1.77-million-compound library in 8.4 h, whereas AutoDock-GPU required an estimated 57.1 days; both runtimes were reported using a single NVIDIA Tesla V100 GPU. This comparison should be interpreted within the specific library, software settings, and hardware configuration used in that study rather than as a universal runtime advantage (Zhang et al., 2023).

### 5.2 Hybrid ML-docking workflows

The most pragmatic role of ML in antiviral VS is as a primary filter. Rather than docking all compounds exhaustively, ML models can eliminate compounds predicted to be inactive, poorly drug-like, chemically unsuitable, or unlikely to interact with the target class of interest. The remaining subset can then undergo docking, rescoring, MD, ADME/Tox filtering, and experimental testing. This filter-first strategy should be distinguished from using ML as a stand-alone oracle. A model may be useful for triage, but it does not directly confirm binding mode, mechanism of action, or antiviral activity (McNutt et al., 2021; Yang et al., 2022; Zhang et al., 2023; Bande and Baday, 2024; Sindt and Rognan, 2026).

### 5.3 ML scoring for dynamic viral targets

A second major application of ML is rescoring. Conventional docking scoring functions are intentionally simplified, making them fast but often inaccurate for ranking binding affinity. ML-based scoring functions learn protein-ligand interaction patterns from datasets of complexes with known structures and experimentally determined affinities. These models can use atom types, contact distances, interaction fingerprints, molecular graphs, 3D grids, protein-ligand distance matrices, or learned embeddings (Yang et al., 2022; Harren et al., 2024).

For polymerase targets, rescoring may be more useful than single-pass docking. A conventional docking program can generate an initial pose ensemble, after which ML models can re-rank poses or compounds. Alternatively, ML models can be applied after MD or ensemble docking to identify compounds whose predicted interactions are robust across receptor conformations. Yet ML rescoring is only as reliable as the data and evaluation framework behind it. If trained primarily on non-viral targets and rigid crystallographic pockets, its predictions for respiratory viral polymerases may fall outside the applicability domain (McNutt et al., 2021; Yang et al., 2022; Zhang et al., 2023; Bande and Baday, 2024; Sindt and Rognan, 2026).

The most realistic near-term approach is hybridization. ML can be applied before docking, after docking, or between multiple structure-based steps. A hybrid RdRp-centered workflow can use ML to filter compounds, docking to generate structural hypotheses, ML rescoring to prioritize robust interactions, MD to assess conformational stability, and experimental assays to validate polymerase inhibition. This iterative logic is preferable to relying on a single computational score to determine antiviral value (McNutt et al., 2021; Yang et al., 2022; Zhang et al., 2023; Bande and Baday, 2024; Sindt and Rognan, 2026).

## 6 Molecular representations for antiviral affinity prediction

ML models operate on representations, not on molecules or proteins as biological entities. The quality, resolution, and biological relevance of these representations determine what a model can learn. For antiviral affinity prediction, the choice of representation determines whether a model captures ligand chemistry, target context, pocket geometry, protein dynamics, nucleotide-like features, and the distinction between viral and non-viral targets (Bouysset and Fiorucci, 2021; Isert et al., 2023; Zhou et al., 2023; Harren et al., 2024).

### 6.1 Descriptors, SMILES and fingerprints

Ligand representations range from simple physicochemical descriptors to high-dimensional graph and three-dimensional encodings. Classical descriptors, including molecular weight, calculated lipophilicity, hydrogen-bond donors and acceptors, TPSA, rotatable bonds, formal charge, aromatic ring count, and molar refractivity, remain useful because they link predictions to medicinal-chemistry constraints. These features can serve as early ADME-aware filters, preventing computational workflows from prioritizing compounds that appear attractive in a pocket but are unlikely to enter cells, remain soluble, or exhibit acceptable drug-like properties (Bouysset and Fiorucci, 2021; Isert et al., 2023; Zhou et al., 2023; Harren et al., 2024).

Fingerprints encode substructures, circular environments, pharmacophores, or molecular fragments. They are efficient for large-scale screening and perform well when chemical similarity to known actives is informative. However, they are limited in their ability to capture three-dimensional complementarity, induced fit, stereochemistry, or the spatial arrangement of contacts within a polymerase active site (Bouysset and Fiorucci, 2021; Isert et al., 2023; Zhou et al., 2023; Harren et al., 2024).

SMILES notation transforms molecular graphs into character strings, allowing natural language processing architectures to be used for molecular graphs. Transformer-based models find this representation advantageous as it helps them learn token dependencies, scaffold patterns, and property-conditioned generation. Nevertheless, SMILES strings can be sensitive to syntax and canonicalization methods, and chemically similar molecules might produce different strings (Bagal et al., 2022).

### 6.2 Graphs, voxels, surfaces and 3D molecular representations

Graph representations are inherently chemical because atoms serve as nodes and bonds as edges. GNNs can incorporate details such as atom identity, valence, hybridization, aromaticity, formal charge, stereochemistry, and bond order (Liao et al., 2019). When applied to protein-ligand systems, graphs may also include covalent ligand structures, residue-level pocket configurations, non-covalent interaction edges, distance-based contacts, and learned message passing across the binding interface (Bouysset and Fiorucci, 2021; Isert et al., 2023; Zhou et al., 2023; Harren et al., 2024).

Three-dimensional representations are central to structure-based ML. Voxel grids encode molecular environments in spatial cells with chemical channels; surface-based representations capture electrostatics, hydrophobicity, curvature, and local shape complementarity; and graph-based representations encode atoms or residues with coordinates and distance-based edges (Zhou et al., 2023). Isert et al. (2023) identified grids, surfaces, and graphs as major macromolecular representations for GDL in structure-based drug design (Isert et al., 2023).

### 6.3 Viral target representations and protein language models

Protein representations hold equal importance. They can include features such as amino acid composition, physicochemical residue descriptors, evolutionary conservation, motifs, and embeddings learned from protein language models (Morehead et al., 2026). These models can produce embeddings that reflect evolutionary and functional patterns, even without full high-resolution structures. This approach is especially useful for viral polymerases, where complete structural data may be lacking, or when comparing related polymerases across different virus families (Bouysset and Fiorucci, 2021; Isert et al., 2023; Zhou et al., 2023; Harren et al., 2024).

For respiratory viral polymerases, relying solely on static ligand descriptors and pocket features is inadequate. A model aimed at identifying RdRp inhibitors should incorporate conformational ensembles, conserved residues involved in contacts, nucleotide-like chemical features, metal-mediated interactions, RNA-adjacent geometry, and ligand classes specific to the mechanism. The choice of representation should therefore be guided by the antiviral objective rather than by software convenience alone (Bouysset and Fiorucci, 2021; Isert et al., 2023; Zhou et al., 2023; Harren et al., 2024).

## 7 Geometric deep learning and pose-generation models

The shift from conventional docking to ML-assisted discovery is most evident in GDL and pose-generation models. Unlike traditional scoring functions, which evaluate candidate poses from heuristic search, GDL models learn from three-dimensional molecular geometry while respecting symmetries such as translation, rotation, reflection, and permutation (Dong et al., 2023). This is attractive for protein-ligand modeling because binding is inherently geometric (Francoeur et al., 2020; Lu et al., 2022; Stärk et al., 2022; Isert et al., 2023; Zhang et al., 2023; Schneuing et al., 2024).

Isert et al. (2023) describe structure-based drug design as a field increasingly shaped by neural network models. These models analyze three-dimensional macromolecular data for tasks like property prediction, binding-site recognition, pose estimation, and *de novo* design. The three main representations used are grids, surfaces, and graphs. Although they differ in how they encode molecular geometry, all these approaches share the goal of making three-dimensional structures accessible to ML algorithms (Isert et al., 2023).

Equivariance is central to this logic. A model is equivariant when input transformations, such as rotation or translation, produce corresponding output transformations. For docking and pose generation, this matters because a ligand-protein complex should not be interpreted differently simply because the coordinate frame has been rotated. EGNNs and related architectures update molecular coordinates or interactions while respecting these symmetries (Francoeur et al., 2020; Lu et al., 2022; Stärk et al., 2022; Isert et al., 2023; Zhang et al., 2023; Schneuing et al., 2024).

EquiBind is an SE (3)-equivariant geometric deep-learning model that predicts the receptor-binding location and ligand pose in a single forward pass, avoiding the iterative sampling used in conventional docking methods. This direct-shot formulation enables rapid blind docking and makes EquiBind attractive for high-throughput screening. However, its fixed-receptor representation does not explicitly model protein conformational adaptation, and the predicted poses may require subsequent geometric or energy-based refinement, particularly for flexible, multidomain targets such as viral polymerases (Stärk et al., 2022; Li et al., 2023).

DiffDock instead formulates molecular docking as a generative diffusion process over the ligand’s translation, rotation, and torsional degrees of freedom. It samples candidate poses through iterative denoising and provides confidence estimates to support pose selection in blind-docking settings. Although DiffDock improves pose-recovery performance relative to several earlier conventional and deep-learning baselines, its standard formulation still treats the receptor conformation as fixed. Consequently, performance may decrease when ligand recognition depends on induced fit, domain motion, or alternative polymerase conformations (Corso et al., 2023; Chen and Zhang, 2025).

KarmaDock summarizes three major categories: models that predict protein-ligand distance matrices and generate poses via optimization; models that use E(n)-equivariant GNN layers to update ligand atom positions; and diffusion-based approaches (Zhang et al., 2023). These approaches share the goal of reducing reliance on slow heuristic sampling while improving pose quality.

KarmaDock is particularly relevant because it integrates protein and ligand encoders, EGNNs with self-attention, post-processing to ensure chemically plausible bond lengths and angles, and a mixture density network to estimate binding strength (Zhang et al., 2023).

In specific evaluation settings, the model reported a docking success rate of 89.1% and a speed of 0.017 s per complex, along with high screening efficiency in an LTK inhibitor discovery project. These data demonstrate technical progress, but LTK is not a viral polymerase, and benchmark success does not automatically establish reliability for RNA-bound, cofactor-dependent viral replication complexes.

For RdRp-centered antiviral discovery, GDL and pose-generation models offer opportunities to accelerate multi-state screening, process structural features simplified by conventional scoring functions, support pocket-guided exploration of binding sites, identify allosteric or interdomain pockets, and integrate MD-derived conformations (Jiang et al., 2025). Yet many models are trained on PDBbind-derived datasets that are unbalanced with respect to viral targets, RNA-containing complexes, polymerase cofactors, or nucleotide-like ligands. This creates a target-domain gap that must be explicitly evaluated (Francoeur et al., 2020; Stärk et al., 2022; Isert et al., 2023; Zhang et al., 2023; Lu et al., 2024; Schneuing et al., 2024).

For this reason, GDL should be incorporated into RdRp discovery as part of an ensemble-aware workflow. The model should not be asked to produce a single best pose for a single receptor. Instead, it should compare poses across biologically defined states, quantify pose stability, identify conserved contacts, and prioritize compounds whose binding hypotheses remain plausible across receptor states (Francoeur et al., 2020; Stärk et al., 2022; Isert et al., 2023; Zhang et al., 2023; Lu et al., 2024; Schneuing et al., 2024).

## 8 Generative AI for antiviral molecule design

### 8.1 From screening existing libraries to generating new chemical space

Conventional VS is constrained by the molecules available in the screened library. Generative AI changes the logic of early discovery by proposing new molecules rather than merely ranking existing ones (Gupta et al., 2018; Bongini et al., 2021; Li et al., 2021). For antiviral discovery, this shift is important because broad-spectrum polymerase inhibitors may require chemotypes that are underrepresented in existing libraries, especially when the goal is to exploit conserved but unusual pockets, nucleotide-like chemistry, or resistance-resistant allosteric mechanisms (Olivecrona et al., 2017; Popova et al., 2018; Brown et al., 2019; Skalic et al., 2019; Ståhl et al., 2019; Bagal et al., 2022; Loeffler et al., 2024).

Skalic et al. (2019) observed that traditional structure-based VS depends on limited compound libraries, which are much smaller than the entire chemical space. Their generative adversarial network was developed to create diverse 3D ligand shapes that fit a protein pocket and decode them into valid SMILES strings. This approach is particularly relevant to antiviral discovery targeting the polymerase because it demonstrates how generative models can be tailored to the target structure rather than to ligand similarity alone (Skalic et al., 2019).

Generative strategies vary by molecular representation and optimization logic. Some models treat molecules as sequences and generate SMILES or SELFIES strings (Arús-Pous et al., 2020; Wei et al., 2023). Others operate on molecular graphs, fragments, scaffolds, or 3D coordinates (Gao et al., 2020; Chen et al., 2023). RL, variational autoencoders, generative adversarial networks, transformers, flow models, and diffusion models have all been applied to molecular design. Each approach offers different trade-offs among validity, novelty, diversity, synthesizability, property control, and structural conditioning (Skalic et al., 2019; Ståhl et al., 2019; Bagal et al., 2022; Loeffler et al., 2024).

### 8.2 Reinforcement learning and multiparameter optimization

RL is attractive for drug design because it enables optimization across multiple objectives. Ståhl et al. (2019) presented a fragment-based actor-critic RL approach for multiparameter optimization. Their case study reported that 93% of the molecules generated were chemically valid and that more than one-third satisfied the targeted objectives, whereas none in the initial set did. In antiviral discovery, this logic is valuable because a polymerase inhibitor must balance potency, selectivity, permeability, metabolic stability, solubility, synthetic tractability, and resistance-barrier penetration (Olivecrona et al., 2017; Popova et al., 2018; Brown et al., 2019; Skalic et al., 2019; Ståhl et al., 2019; Bagal et al., 2022; Loeffler et al., 2024).

### 8.3 Transformers and molecular language models

Transformers and molecular language models offer another approach. Bagal et al. (2022) introduced MolGPT, a transformer-decoder model trained to predict the next token in molecular SMILES strings. MolGPT generated valid, unique, and novel drug-like molecules and could be trained conditionally to control multiple molecular properties and scaffold constraints (Bagal et al., 2022). This is relevant to antiviral design because scaffolds associated with nucleoside analogs, non-nucleoside inhibitors, or known polymerase-binding motifs can serve as constraints in optimizing physicochemical properties.

### 8.4 REINVENT 4 and open-source generative design

REINVENT 4 provides an operational example of modern generative design software. This framework builds on earlier REINVENT releases that established reinforcement-learning-based molecular optimization (Blaschke et al., 2020). Loeffler et al. (2024) describe REINVENT 4 as an open-source generative AI framework that uses recurrent neural networks and transformer architectures, embedded within transfer learning, RL, and curriculum-learning workflows. The framework supports *de novo* design, R-group replacement, library and linker design, scaffold hopping, and molecule optimization (Loeffler et al., 2024).

Tirosyan et al. (2026) offer an antiviral-specific perspective and summarize emerging examples of AI-guided antiviral design. Peer-reviewed primary evidence provides proof of concept that generative models can move beyond *in silico* nomination: Izmailyan et al. (2024) reported influenza neuraminidase inhibitors generated through a reinforcement-learning workflow and evaluated them *in vitro* and *in vivo*. Nevertheless, the number of antiviral candidates supported by comparable experimental validation remains limited, and generative outputs should be regarded as testable chemical hypotheses unless accompanied by synthesis and orthogonal biological validation (Izmailyan et al., 2024; Tirosyan et al., 2026).

### 8.5 Why generative validity is not antiviral validity

The key caution is that generative validity differs from antiviral validity. Tirosyan et al. (2026) explicitly state that high computational scores and molecular novelty do not guarantee biological effectiveness, as factors such as solubility, cell permeability, metabolic stability, and cytotoxicity significantly influence *in vitro* and *in vivo* outcomes. Without experimental validation, outputs from generative AI are merely hypotheses (Tirosyan et al., 2026).

## 9 Benchmarking ML models for protein-ligand affinity prediction

Benchmarking marks the point at which computational promise becomes measurable. For ML-based protein-ligand affinity prediction, benchmarking can assess regression accuracy, active-decoy discrimination (Mysinger et al., 2012), binding-pose recovery, early enrichment, or prospective hit prioritization. These tasks are related but not equivalent. A model that reproduces retrospective affinity labels may still fail at pose generation, prospective virtual screening, viral-target transfer, or antiviral activity prediction. For RdRp-centered discovery, the benchmark must therefore be interpreted as part of a pharmacological decision framework rather than as a purely computational leaderboard (Liu et al., 2017; Su et al., 2019; Harren et al., 2024; Landrum and Riniker, 2024).

### 9.1 Scope and literature-search strategy

This narrative review was supported by a structured literature search rather than conducted as a systematic review or meta-analysis. PubMed and Google Scholar were searched for publications through August 2026. Search terms were organized around four domains: (i) artificial intelligence, machine learning, deep learning, geometric deep learning, generative models, molecular docking, and binding-affinity prediction; (ii) antiviral discovery and respiratory RNA viruses; (iii) RNA-dependent RNA polymerase, RdRp, nsp12, PB1, and mononegavirus L protein; and (iv) biochemical validation, cellular antiviral activity, cytotoxicity, resistance, pharmacokinetics, and ADME/Tox. Representative combinations included (“artificial intelligence” OR “machine learning” OR “deep learning” OR docking) AND (antiviral OR “respiratory virus”) AND (RdRp OR “RNA-dependent RNA polymerase” OR nsp12 OR PB1 OR “L protein”), together with targeted searches for protein–ligand affinity benchmarking using RMSE and related evaluation metrics.

Peer-reviewed primary studies were prioritized to support mechanistic, structural, computational, biochemical, cellular, resistance, or pharmacological claims. Recent reviews were used to contextualize broader developments and identify additional primary studies by screening reference lists. Earlier foundational publications were retained when necessary to describe polymerase architecture, established computational methods, benchmark datasets, or validated antiviral mechanisms. Studies were considered for inclusion if they addressed AI-guided antiviral discovery, respiratory viral polymerases, transferable protein–ligand modeling methods, or experimental validation relevant to the proposed pipeline. Studies focused exclusively on diagnostic or prognostic AI applications, records lacking a verifiable publication source, duplicate reports, and articles without a clear connection to antiviral drug discovery were excluded.

Preprints and early reports were used only to describe emerging directions, were clearly identified as such, and were not used as the sole basis for claims of experimental or clinical validation. Peer-reviewed online-ahead-of-print articles were retained when their publication status and DOI could be verified. General protein–ligand benchmarking studies were included for their methodological relevance but were classified as indirect evidence unless they explicitly evaluated viral targets or RdRp-containing complexes. Study selection for the broader review was therefore guided by relevance to the analytical framework rather than by an intention to conduct an exhaustive quantitative synthesis.

Within this broader narrative search, a separate, targeted selection process was conducted for the recent ML and DL benchmarking studies summarized in Table 1. This focused search covered publications from January 2024 through August 2026 and prioritized peer-reviewed studies reporting RMSE or related quantitative metrics for protein–ligand binding-affinity prediction. The corresponding search strings, record-screening stages, eligibility assessment, and the final number of included studies are presented in the PRISMA flow diagram in Figure 3. Figure 3 therefore documents the reproducible selection of the benchmarking subset used in Table 1 and should not be interpreted as representing the entire narrative evidence base of the review.

**TABLE 1:** Benchmarking landscape of recent ML and DL models for protein-ligand binding-affinity prediction.

​	Study	Model/architecture	Representation	Reported RMSE (affinity label/scale)	Dataset/benchmark	Evaluation context	Viral/RdRp relevance	Interpretation for RdRp discovery
A. CASF-2016-centered standardized benchmark evaluations	(Mohamed Abdul Cader et al., 2024)	DL with self-attention and cross-attention	Structural and sequence features	0.957 (CASF affinity pK; pKd/pKi)	CASF-2016 and related benchmarks	CASF-2016-centered evaluation	No direct viral or RdRp target reported	General affinity benchmark; RdRp transferability remains untested
	(Rahman et al., 2024)	Attention-based DL ensemble	Atomic distances and amino-acid sequences	0.987–1.061 (dataset-specific pK; CASF pKd/pKi and CSAR-HiQ)	CASF-2016 and related benchmarks	CASF-centered multi-benchmark evaluation	No direct viral or RdRp target reported	Useful for general scoring comparison, but not evidence of antiviral relevance
	(Lodh et al., 2026)	ResNet plus LSTM	Physicochemical descriptors and interaction energies	0.993 (CASF affinity pK; pKd/pKi)	CASF-2016	CASF-2016 benchmark evaluation	No direct viral or RdRp target reported	Hybrid features require RdRp-specific testing
	(Lodh et al., 2025)	1D CNN plus GRU	Numeric descriptors, amino-acid counts, and protein sequences	1.001–1.003 (PDBbind pK and CASF pKd/pKi)	PDBbind v2020; CASF-2016	PDBbind/CASF benchmark evaluation	No direct viral or RdRp target reported	Competitive general performance, but no RNA-bound polymerase validation
	(Rahman et al., 2026)	Angle-Aware Predictor	Dihedral-angle features	1.072 (CASF affinity pK; pKd/pKi)	CASF-2016	CASF-2016 benchmark evaluation	No direct viral or RdRp target reported	Applicability to flexible RdRp active sites remains prospective
	(Sim and Lee, 2026)	GatedGCN-LSPE	Protein–ligand graphs	1.100 (CASF affinity pK; pKd/pKi)	CASF-2016	CASF-2016 benchmark evaluation	No direct viral or RdRp target reported	Requires external testing on polymerase complexes
B. General PDBbind and multi-benchmark evaluations	(Lim et al., 2025)	CheapNet: GNN encoder, differentiable pooling, and cross-attention	3D molecular graphs with atomic coordinates and interaction edges	1.107–1.343 (PDBbind pK and CASF pKd/pKi)	PDBbind; CSAR NRC-HiQ; CASF-2016; DUD-E	Multi-benchmark affinity and screening evaluation	No direct viral or RdRp target reported	Insufficient for RdRp prioritization without target-family validation
	(Li et al., 2026b)	HybridGeo: geometric DL and graph transformer	2D/3D graphs and residue-scale pocket graphs	1.172 (PDBbind composite pK)	PDBbind and external test sets	PDBbind plus external evaluation	No direct viral or RdRp target reported	Relevant geometric framework, but RdRp transferability remains untested
	(Wang et al., 2024)	Dual transformer encoders plus GIN	Protein/pocket sequences and 2D ligand graphs	1.203–1.388 (PDBbind composite pK)	PDBbind v2020	PDBbind benchmark evaluation	No direct viral or RdRp target reported	Viral-polymerase applicability requires external validation
	(Song et al., 2025)	NciaNet interaction-aware graph network	3D complexes focused on non-covalent interactions	1.208–1.409 (PDBbind composite pK)	PDBbind	PDBbind benchmark evaluation	No direct viral or RdRp target reported	May omit RNA, metals, and cofactors unless explicitly represented
	(Li et al., 2026b ; Li et al., 2025)	PLGAs: GCN plus global attention mechanism	3D protein–ligand complex graphs	1.211 kcal/mol, as reported by the authors	PDBbind v2019 refined and v2016 core sets	PDBbind benchmark evaluation	No direct viral or RdRp target reported	Source-reported scale is not directly comparable with pK-based RMSE values
	(Mahdizadeh and Eriksson, 2025)	iScore-Hybrid: DNN, RF, and XGBoost	Ligand and binding-pocket descriptors	1.23–1.34 (PDBbind pK and CASF pKd/pKi)	PDBbind 2020; CASF-2016	PDBbind/CASF benchmark evaluation	No direct viral or RdRp target reported	Requires polymerase-specific applicability assessment
	(Huang et al., 2025b)	Multidimensional CNN/MCDTA	1D sequence, 2D structure, and 4D pocket tensor	1.231 (PDBbind composite pK)	PDBbind v2020	PDBbind benchmark evaluation	No direct viral or RdRp target reported	No direct evidence of RdRp performance
	(Liu and Pan, 2025)	Dual-gated dynamic interaction GNN	3D interaction graph with RDKit and RBF features	1.252–1.395 (PDBbind pK and CASF pKd/pKi)	PDBbind; CASF; temporal holdout	Evaluation including temporal holdout	No direct viral or RdRp target reported	Temporal testing improves rigor, but polymerase generalization remains unproven
	(Liu et al., 2026a)	AAPPE deep-learning framework	Amino-acid pair encoding and ligand fingerprints	1.280 (CASF affinity pK; pKd/pKi)	CASF-2016	CASF-2016 benchmark evaluation	No direct viral or RdRp target reported	No RdRp-specific target coverage reported
	(Huang et al., 2025a)	Invariant heterogeneous geometric GNN	3D ligand/pocket graphs with directional geometric features	1.291–1.317 (PDBbind composite pK)	PDBbind v2013, v2016, and v2019	Evaluation across multiple PDBbind versions	No direct viral or RdRp target reported	Requires testing on metal-dependent polymerase pockets
	(Yoon et al., 2026)	ESM2, Chemformer, Bi-LSTM, transformers, and cross-attention	Protein/pocket sequences, ligand SMILES, and 2D graphs	1.304–1.469 (mixed affinity labels)	PDBbind v2016; BindingDB; CASF-2013	Multi-source benchmark evaluation	No direct viral or RdRp target reported	Target-aware representation, but RdRp relevance remains inferential
	(Rayka et al., 2025)	2D CNN variants	Inverse-distance interatomic contact matrix	1.353–1.471 (PDBbind composite pK)	PDBbind 2016	PDBbind benchmark evaluation	No direct viral or RdRp target reported	No direct validation in viral-polymerase systems
	(Li et al., 2026c)	FlowDock: flow-based model with SE (3)-equivariant modules	ESM2 embeddings, 3D proteins, ligand SMILES, and graphs	1.413 (PDBbind/BindingDB composite pK)	PDBbind; PoseBusters; DEKOIS 2.0; BindingDB	Pose, affinity, and decoy evaluation	No direct viral or RdRp target reported	Requires RdRp-specific benchmarking before use as antiviral evidence
C. Viral-target or non-standard challenge evaluations	(Krishnan and Vinod, 2024)	GCCR graph-convolutional capsule regression	Chemical graphs	0.0978–0.3806 (non-standard internal/Davis-derived scales)	Davis dataset	Kinase-style benchmark with indirect SARS-CoV-2 filtering	Indirect antiviral relevance; no direct RdRp benchmark	Not comparable with PDBbind/CASF pK-based RMSE values
	(Liu et al., 2026b)	LRIP-SF with MM-GBSA decomposition	Ligand–residue interaction profiles	0.813–0.894 (pIC50)	ASAP Antiviral Challenge 2025; SARS-CoV-2/MERS Mpro	Antiviral protease challenge evaluation	Direct viral-target relevance, but not RdRp-specific	Findings should not be generalized to RdRp without polymerase testing
	(Ranganath et al., 2026)	Multimodal graph network with EGNN/SGNN	3D molecular graphs with atomic features	1.288 (PDBbind composite pK)	PDBbind 2020; flavivirus; SARS-CoV-2 Mpro	General benchmark plus viral-target evaluations	Direct viral-target evaluation, but no direct RdRp testing	Antiviral-proximal evidence; polymerase-specific validation remains necessary

Evaluation groups: A, CASF-2016-centered standardized benchmark evaluations; B, general PDBbind and multi-benchmark evaluations; C, viral-target or non-standard challenge evaluations. RMSE values should only be compared when the affinity definitions, units, datasets, split strategies, and test sets are equivalent.

Rows are grouped by evaluation context to avoid implying that all RMSE values are directly comparable. RMSE values should be interpreted only within matched datasets, endpoints, units, test sets, and split strategies. Transferability to respiratory viral polymerases depends on target coverage, dataset composition, receptor state, and the context of experimental validation.

**FIGURE 3:**
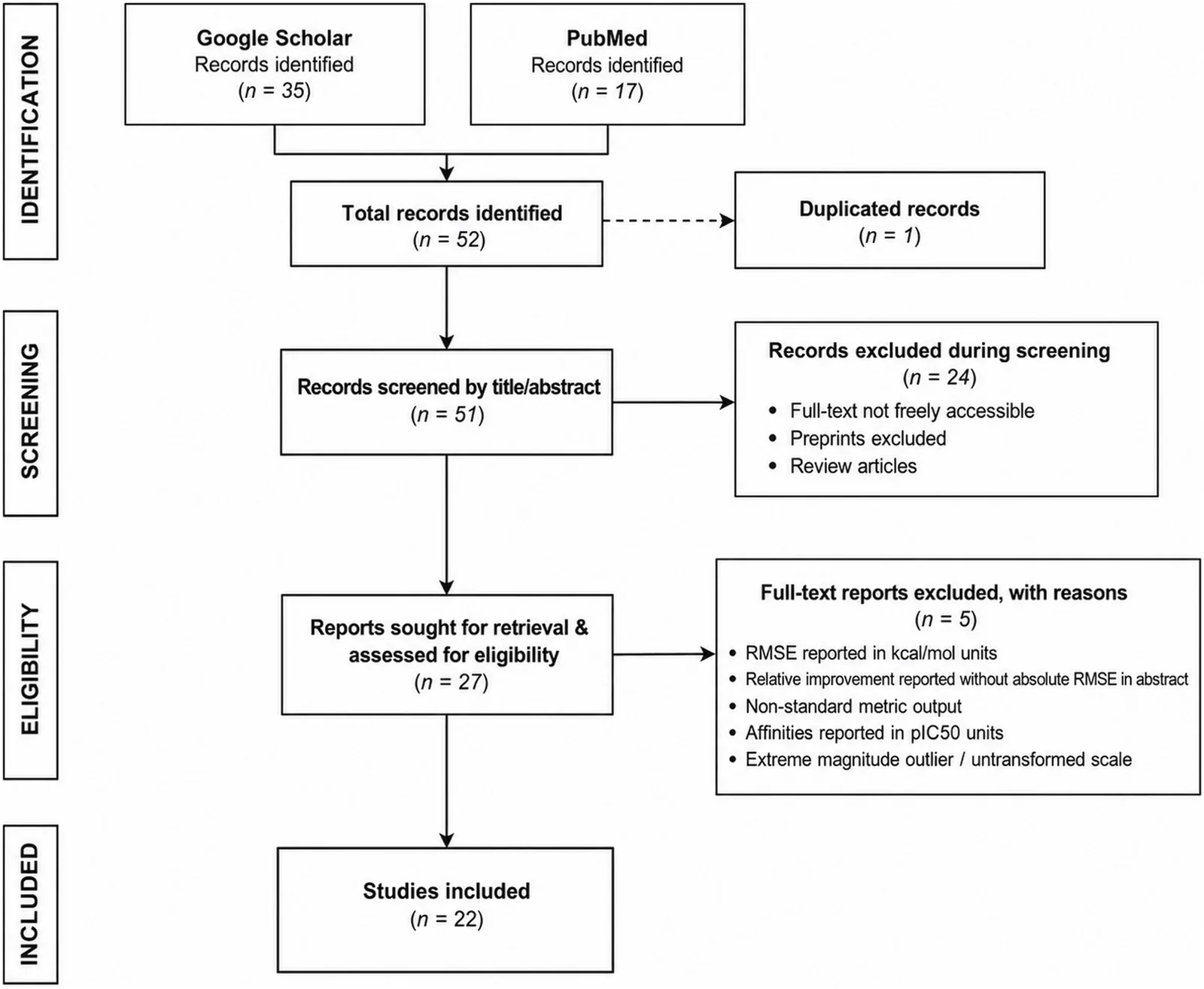
PRISMA flow diagram for the targeted selection of benchmarking studies included in Table 1. The diagram summarizes the focused search conducted in PubMed and Google Scholar from January 2024 through August 2026 to identify peer-reviewed studies reporting machine-learning or deep-learning performance for protein–ligand binding-affinity prediction. The PubMed search string was *(“machine learning” OR “deep learning”) AND “protein-ligand” AND “binding affinity” AND (“RMSE” [All Fields] OR “root mean squared error” [All Fields])*, whereas the Google Scholar search was *intitle: “protein-ligand” intitle: “binding affinity” (“machine learning” OR “deep learning”) “RMSE” - “review”*. This PRISMA diagram represents only the benchmarking subset summarized in Table 1 and not the broader narrative literature base used throughout the review.

### 9.2 What RMSE means and what it does not mean

RMSE is the square root of the mean squared difference between predicted and experimental affinity values. In protein-ligand affinity prediction, it is typically reported in log-transformed units such as pKd, pKi, pIC50, or pK. A lower RMSE indicates closer agreement with the benchmark labels, but only for the specific label set and evaluation protocol used in the study. An RMSE near one log unit can correspond to an average error of approximately one order of magnitude in affinity, which is pharmacologically meaningful during hit-to-lead prioritization (Liu et al., 2017; Harren et al., 2024; Landrum and Riniker, 2024).

For antiviral discovery, RMSE is informative yet incomplete. It does not capture early enrichment in a prospective screen, plausibility, target engagement, cellular uptake, intracellular activation, cytotoxicity, resistance-barrier penetration, or potency in infected cells. This distinction is central to the present review. A model with an attractive RMSE on CASF or PDBbind may be useful as a triage tool, but it cannot be taken as evidence that a compound will inhibit a respiratory viral polymerase in infected cells (Smee et al., 2017; Su et al., 2019; Von Delft et al., 2023; Tirosyan et al., 2026).

### 9.3 Lessons from the benchmarking table

Table 1 highlights three conclusions relevant to RdRp-centered antiviral discovery. First, most recent models rely on DL architectures, including GNNs, geometric message passing, transformers, attention-based encoders, convolutional models, recurrent sequence models, and hybrid ensembles. Second, input representations vary widely, ranging from one-dimensional sequences and SMILES strings to ligand graphs, residue-level pocket graphs, three-dimensional complex graphs, distance matrices, non-covalent interaction fingerprints, and hybrid energetic descriptors. Third, direct viral-target evaluation remains uncommon; most studies are still trained and validated on general datasets such as PDBbind and CASF (Liu et al., 2017; Su et al., 2019; Harren et al., 2024).

Importantly, the studies summarized in Table 1 should not be considered equivalent in terms of their relevance to respiratory viral RdRp. This distinction also highlights an important and still underexplored area of research, with substantial potential for further investigation in the coming years. To clarify this issue, we categorize the available evidence according to its biological proximity to the intended application. The highest level of relevance comprises models evaluated directly on respiratory viral polymerases or RdRp-containing complexes. A second category includes models evaluated on viral proteins or antiviral datasets, but not specifically on RdRp. These studies provide evidence of potential transferability to viral targets, but do not constitute direct evidence of polymerase-specific performance. The third category comprises models evaluated primarily using general protein–ligand benchmarks, including PDBbind and CASF. These studies provide valuable information regarding general affinity-prediction capability and contribute to the optimization of molecular representations and model architectures; however, their reported performance should not be interpreted as direct evidence of applicability to respiratory viral RdRp. (Su et al., 2019; Peele et al., 2020; Landrum and Riniker, 2024; Graber et al., 2025; Li et al., 2026a).

This distinction is particularly important when comparing performance metrics reported across different studies. A low RMSE obtained on a general benchmark does not necessarily imply superior performance on a respiratory viral polymerase, because the underlying target distributions, structural flexibility, ligand chemotypes, experimental endpoints, and levels of protein–ligand redundancy may differ substantially across datasets. Conversely, a model showing less favorable performance on a general benchmark may have greater biological relevance if it has been evaluated on viral polymerases or antiviral datasets. Therefore, general protein–ligand benchmarks are interpreted primarily as evidence of methodological capability, whereas stronger claims regarding RdRp relevance are reserved for studies that include direct evaluation on viral polymerases.

## 10 Dataset bias, leakage and viral-target transferability

Modern ML models can appear highly accurate when trained and evaluated on datasets with hidden redundancy, similar ligands, related protein families, or comparable pocket structures across training and test splits. If a model performs well because it has already seen close neighbors of the test examples, its apparent generalization may not hold for a new viral polymerase target, an RNA-bound conformation, or a chemically distinct inhibitor series (Liu et al., 2017; Su et al., 2019; Landrum and Riniker, 2024; Graber et al., 2025; Li et al., 2026a).

PDBbind and CASF are foundational resources for protein-ligand modeling. PDBbind provides experimentally measured binding data for protein-ligand complexes with resolved structures, and CASF provides standardized tasks for scoring, ranking, docking, and screening power (Liu et al., 2017; Su et al., 2019). However, these resources are built around complexes with resolved structures, which means they overrepresent experimentally tractable and historically studied targets and chemotypes. Viral polymerases, especially those associated with dynamic RNA-associated respiratory virus complexes, are not a dominant class.

Bias enters at multiple levels. Target bias arises when certain protein families are overrepresented. Chemical bias arises when ligands cluster around familiar scaffolds. Structural bias arises when similar pockets or homologous proteins appear in both the training and test sets. Label bias arises when different experimental measurements (Kd, Ki, IC_50_, EC_50_, or other endpoints) are aggregated without careful context. Methodological bias arises when model selection repeatedly exploits the same benchmark (Liu et al., 2017; Su et al., 2019; Landrum and Riniker, 2024; Graber et al., 2025; Li et al., 2026a).

Leakage is a particularly serious form of bias. It occurs when information from the training set indirectly appears in the test set through similar ligands, homologous proteins, or nearly identical binding pockets. Recent studies have shown that removing redundancy and using stricter splits can substantially reduce apparent model performance, indicating that some reported gains reflect dataset structure rather than true extrapolative ability (Graber et al., 2025; Li et al., 2026a). For antiviral polymerases, leakage-resistant evaluation is essential.

Label heterogeneity is another major issue. BindingDB and related resources aggregate measurements from diverse assays, conditions, proteins, constructs, and experimental formats (Liu et al., 2007; Gilson et al., 2016). This breadth is valuable, but combining Kd, Ki, IC_50_, and EC_50_ values indiscriminately can obscure the learning target. In antiviral discovery, target-level affinity, enzyme inhibition, and cell-based antiviral potency are distinct biological quantities (Liu et al., 2017; Su et al., 2019; Landrum and Riniker, 2024; Graber et al., 2025; Li et al., 2026a).

An RdRp-centered benchmark should therefore be task-specific and biologically stratified. It should specify the task as affinity regression, active/inactive classification, pose generation, early enrichment, cross-virus generalization, resistance-aware prediction, or cell-based antiviral prioritization. It should split the data by target family, viral species, receptor conformation, ligand scaffold, and mechanism of inhibition. It should report performance separately for viral and non-viral targets, nucleoside and non-nucleoside inhibitors, catalytic and allosteric sites, and biochemical versus cellular endpoints (Liu et al., 2017; Su et al., 2019; Landrum and Riniker, 2024; Graber et al., 2025; Li et al., 2026a).

## 11 Current AI applications in antiviral discovery against RNA viruses

AI applications in antiviral discovery span multiple levels of biological proximity. Some studies identify host or viral targets using omics data, protein-protein interaction data, or genetic screens. Others prioritize approved drugs or bioactive compounds for repurposing. A third group uses structure-based ML, docking, or generative models to propose small-molecule inhibitors. A fourth area predicts viral mutations and resistance effects from sequence data. Tirosyan et al. (2026) organize the antiviral AI landscape around target identification, repurposing, *de novo* molecule design, and resistance prediction (Tirosyan et al., 2026). These applications and their corresponding levels of experimental evidence are summarized in Table 2.

**TABLE 2:** Evidence-level synthesis of AI-guided antiviral discovery studies, organized by experimental proximity rather than as a flat catalog.

Evidence level and representative AI strategy	Concrete examples and targets	Main limitations in the RdRp context	Recommended minimum reporting standards
In silico-only prioritization: drug-target interaction prediction, network-based repurposing, and ML-assisted virtual screening	Ultra-large docking against SARS-CoV-2 targets, including RdRp; norovirus RdRp drug-prioritization workflows combining ML, docking, and molecular dynamics; ZINC20-scale virtual screening campaigns	High docking or ML scores do not demonstrate antiviral activity. These workflows often omit intracellular exposure, active metabolite formation, cytotoxicity, and liability to resistance.	Report the target structure or model, receptor state, ligand form, training data, split strategy, applicability domain, uncertainty estimates, controls, scoring protocol, and the rule used to select top candidates. Avoid describing predicted affinity as antiviral efficacy.
Target identification: host-virus protein interactions, omics-based ranking, and CRISPR interpretation	Viral dependency-factor screens, host-directed antiviral networks, and pathway-level prioritization for respiratory viruses	Biological essentiality is not equivalent to structural druggability. Host-directed targets may show strong genetic signals but still carry toxicity, redundancy, or cell-context dependence.	Report virus, strain or variant, cell model, perturbation method, effect size, replication, host-cell viability, and orthogonal validation of target relevance and druggability. Distinguish direct-acting viral targets from host-directed targets.
Structure-based ML and pose generation: geometric deep learning, equivariant GNNs, and diffusion-based pose prediction	KarmaDock, EquiBind, DiffDock, and models benchmarked on PDBbind/CASF-type datasets	Models trained on generic protein-ligand complexes may generalize poorly to RNA-bound, metal-dependent viral polymerases. RNA templates, divalent metals, catalytic motifs, and cofactors such as nsp7/nsp8 or RSV P protein are often underrepresented.	Report PDB identifiers or model sources, polymerase state, RNA presence, metal ions, cofactors, missing regions, protonation states, binding-site definition, ensemble strategy, positive and negative controls, and pose-confidence criteria.
Generative AI and *de novo* design: reinforcement learning, transformers, generative adversarial models, and scaffold-conditioned generation	REINVENT 4, MolGPT, DS-22-inf-009/021 for influenza neuraminidase, and Chemistry42-derived SARS-CoV-2 Mpro candidates such as ISM3312	Chemically valid molecules may still be synthetically difficult to prepare, metabolically unstable, cytotoxic, or inactive in cells. For nucleoside analogs, parent compounds may not generate the active intracellular triphosphate form required for RdRp recognition.	Report novelty, synthetic accessibility, structural alerts, predicted ADME/Tox, off-target liabilities, uncertainty, parent-drug versus active-metabolite representation, and experimental confirmation of identity and purity after synthesis.
Biochemical and cellular validation of prioritized candidates	Recombinant RdRp primer-extension assays, minigenome systems, infected-cell EC_50_ assays, CC_50_ cytotoxicity testing, and remdesivir as a mechanistic nsp12 reference compound	Biochemical inhibition may not translate into cellular antiviral activity due to uptake, metabolism, or toxicity. Reduced viral RNA or reporter signal alone may not confirm inhibition of infectious-virus production.	Report enzyme complex composition, RNA template, nucleotide and metal conditions, concentration-response curves, IC50 or Ki, positive controls, virus strain, cell model, MOI, treatment timing, antiviral readout, EC_50_, CC_50_ measured in parallel, selectivity index, infectious-yield confirmation, and biological replicates.
*In vivo*-supported candidates: AI-prioritized or generative molecules followed by animal efficacy testing	DS-22-inf-009 protective-efficacy studies in influenza mouse models and clinical-stage progression reported for AI-supported coronavirus protease candidates	Cellular potency does not guarantee therapeutic exposure, tissue penetration, tolerability, or protection *in vivo*. Viral load reductions must be interpreted alongside pharmacokinetic and toxicity data.	Report animal model, route, dose, schedule, randomization or blinding when applicable, pharmacokinetic exposure relative to cellular potency, tissue viral titers or infectious yield, clinical or survival endpoints, and tolerability.
Resistance-aware prediction: sequence-based models, mutation-effect prediction, and variant-surveillance integration	RNA-virus escape tracking, mutation-effect prediction, and RdRp substitutions affecting nucleoside-analog susceptibility, including SARS-CoV-2 nsp12 resistance-relevant sites	Computational mutation-effect predictions cannot establish resistance barriers or fitness costs without experimental testing. Single substitutions may behave differently across viral backgrounds.	Report mutation identity, selection or engineering method, sequencing confirmation, fold-change in biochemical or cellular susceptibility, viral fitness cost, passage history, and whether resistance data are fed back into the optimization cycle.

Concrete examples, key limitations, and recommended minimum reporting standards are included to distinguish computational prioritization from experimentally supported antiviral evidence.

### 11.1 *In silico*-only and repurposing studies

Drug repurposing was among the earliest and most visible AI applications during the COVID-19 pandemic (Cousins et al., 2024). ML and DL models were used to predict drug-target interactions, map host-virus networks, analyze transcriptomic signatures, and prioritize approved or investigational compounds (Jo et al., 2022). For respiratory virus research, repurposing is attractive because safety, formulation, and pharmacokinetic data may already be available. However, repurposing predictions should be interpreted with pharmacological realism in mind, including tissue distribution, intracellular concentration, protein binding, and cytotoxicity (Zhu et al., 2020; Tirosyan et al., 2026).

Target-based antiviral AI has often focused on well-resolved viral proteins, especially the SARS-CoV-2 main protease, papain-like protease, spike protein, and RdRp. These targets were selected because they are essential, structurally characterized, and amenable to experimental assays. For polymerase discovery, AI has been used to prioritize inhibitors, predict drug-target interactions, and identify chemical libraries enriched for antiviral activity (Zhu et al., 2020; Tirosyan et al., 2026).

### 11.2 *In vitro*-validated studies

Generative AI has produced some of the most prominent recent examples. Tirosyan et al. (2026) summarize RL-based generative design of the influenza neuraminidase inhibitors DS-22-inf-009 and DS-22-inf-021, which reportedly showed *in vitro* activity against influenza A and B strains, with EC_50_ values ranging from 0.29 to 2.31 µM, and *in vivo* protective efficacy in mice. They also discuss ISM3312, a SARS-CoV-2 main protease inhibitor generated using Chemistry42, which has been reported to have activity against multiple human coronaviruses and has completed a Phase I study in China (Tirosyan et al., 2026).

### 11.3 *In vivo*-supported and challenge-based evidence

The Billion Molecules against COVID-19 Challenge offers another important lesson. AI-selected molecules achieved a higher experimental hit rate than those selected primarily through traditional VS and docking, according to the reported comparison (Tirosyan et al., 2026). However, hit rate does not equal clinical relevance. The practical lesson is that AI can improve enrichment when embedded in a well-defined challenge with consistent targets, filtering criteria, and biological assays.

### 11.4 Lessons for respiratory RNA virus polymerases

Based on the currently available peer-reviewed literature, AI applications for RdRp-centered discovery remain fragmented. SARS-CoV-2 RdRp has attracted docking, MD, and ML-assisted screening studies, although a substantial proportion of these studies remain predominantly *in silico*. Influenza polymerase serves as a clinically validated target node, yet dedicated AI benchmarking for this polymerase remains comparatively limited. For RSV L and related mononegavirus polymerases, the available structural and ligand datasets remain smaller than those for more extensively studied antiviral targets (Zhu et al., 2020; Tirosyan et al., 2026).

## 12 Experimental translation: from predicted affinity to antiviral activity

The translational value of AI-guided antiviral discovery depends on whether computational predictions identify compounds that are effective in biological systems. For RdRp-centered discovery, this requires a validation cascade that separates predicted binding, physical target engagement, biochemical polymerase inhibition, cell-based antiviral activity, cytotoxicity, resistance barrier, and *in vivo* feasibility. These levels are related but not interchangeable (Smee et al., 2017; Zhu et al., 2020; Von Delft et al., 2023; Tirosyan et al., 2026).

The first experimental layer is target-proximal validation. For polymerase inhibitors, this may include assays with purified polymerase, primer extension, nucleotide incorporation, RNA synthesis, capping or methyltransferase assays for L proteins, minigenome systems, or reporter-based polymerase activity assays. These systems test whether the compound interferes with the proposed viral replication machinery. Their advantage is mechanistic clarity, but they may not fully replicate the infected-cell context (Smee et al., 2017; Zhu et al., 2020; Von Delft et al., 2023; Tirosyan et al., 2026).

Implementing these target-proximal assays is not trivial. Reconstituting an active respiratory viral polymerase may require the coordinated expression and purification of catalytic subunits, accessory cofactors, RNA templates, and metal ions under conditions that preserve the correct stoichiometry and conformational state. This complexity can limit protein yield, reproducibility, assay throughput, and interlaboratory comparability, particularly for large mononegavirus L–P complexes and multiprotein coronavirus replication systems. Minigenome and polymerase-reporter assays partially overcome these limitations by measuring intracellular polymerase-dependent activity without requiring the complete infectious cycle, thereby improving scalability and reducing biosafety requirements. However, these systems do not recapitulate viral entry, genome packaging, assembly, release, or the full influence of other viral proteins and host responses. In contrast, infectious-virus assays provide the most biologically integrated measure of antiviral activity but require suitable containment facilities, trained personnel, validated cell models, and more resource-intensive workflows. Consequently, activity detected in a purified-polymerase or minigenome assay should be confirmed using infectious-virus or orthogonal cellular assays when claims concern antiviral efficacy rather than isolated polymerase inhibition (Te Velthuis and Fodor, 2016; Zhu et al., 2020; Malone et al., 2022; Bonneux et al., 2024).

The second layer is physical or cellular target engagement. Binding can be assessed using surface plasmon resonance, isothermal titration calorimetry, thermal shift assays, differential scanning fluorimetry, chemical proteomics, photoaffinity labeling, or cellular thermal shift assays. For viral polymerases, target engagement can be technically challenging because the active complex may require RNA, cofactors, or viral infection. Nevertheless, orthogonal evidence of engagement is important if a manuscript claims a direct polymerase mechanism (Molina et al., 2013; Schenone et al., 2013; Savitski et al., 2014; Zhu et al., 2020).

The third layer is cell-based antiviral activity. These assays assess whether the compound reduces viral replication, viral RNA, infectious virus production, antigen expression, cytopathic effect, or reporter signal in infected cells (Praditya et al., 2025). However, cell-based antiviral activity alone does not establish the target mechanism. A compound may reduce viral readouts by impairing entry, translation, host metabolism, cell viability, or assay signal. Therefore, antiviral assays should be paired with cytotoxicity assays and, when possible, time-of-addition experiments, polymerase reporter systems, or resistance mapping (Smee et al., 2017; Zhu et al., 2020; Von Delft et al., 2023; Tirosyan et al., 2026).

For nucleoside analogs, the validation cascade requires additional layers. Activity depends on cellular uptake, phosphorylation, triphosphate formation, competition with endogenous nucleotides, incorporation by viral polymerase, and the resulting termination or mutagenesis. A compound docked as a parent nucleoside may not represent the active species; a compound docked as a triphosphate may not be generated efficiently in cells (Zhu et al., 2020; Vicenti et al., 2021; Xu et al., 2022).

PK/PD and ADME considerations should be addressed earlier than many computational studies acknowledge. A compound that inhibits viral polymerase in cells at µM concentrations may still fail if exposure in respiratory tissues is inadequate or toxicity limits dosing. AI pipelines should incorporate solubility, permeability, metabolic stability, plasma protein binding, transporter liability, and predicted tissue exposure as prioritization features rather than as late-stage exclusions (Smee et al., 2017; Zhu et al., 2020; Von Delft et al., 2023; Tirosyan et al., 2026).

It is important to recognize that experimental translation encompasses both dry-lab and wet-lab components, each of which entails independent and potentially substantial costs during antiviral drug discovery. Although AI-guided approaches are intended to increase efficiency by reducing the time and experimental burden associated with developing and prioritizing new compounds, computational predictions ultimately require experimental confirmation via conventional validation workflows (Roney and Mohd Aluwi, 2024; Bhat and Ahmed, 2025).

The feasibility and reproducibility of this approach also depend on access to adequate computational infrastructure and specialized personnel. Although several publicly available platforms and resources that incorporate AI-based methods, such as DeepChem and AlphaFold, can reduce some computational and implementation costs, the wet-lab validation of *in silico* predictions remains a major practical challenge. Experimental validation may require access to appropriate cell lines, culture media, specialized equipment, molecular reagents, and facilities capable of handling the relevant respiratory viruses under appropriate biosafety conditions. These requirements can substantially increase both the cost and logistical complexity of the validation process. Consequently, although the expansion of pharmaceutical research and the SARS-CoV-2 pandemic contributed to increased investment in antiviral research and biosafety infrastructure, access to the facilities, expertise, and resources required to implement AI-guided antiviral discovery remains heterogeneous across institutions and geographic regions. This variability in infrastructure represents an important consideration when assessing the real-world feasibility and scalability of AI-guided strategies for respiratory antiviral discovery (Ta et al., 2019; Chakraborty et al., 2024). Network-driven initiatives such as the Red Mexicana de Desarrollo de Antivirales (RMDA) illustrate how computational prioritization, BSL-2/BSL-3 antiviral testing, mechanistic virology, synergy assessment, *in vivo* validation, and clinical linkage can be organized within a regionally adaptable translational framework (De Jesús-González et al., 2026).

## 13 Proposed RdRp-centered AI pipeline for broad-spectrum respiratory antivirals

The preceding sections identify a methodological gap: AI-guided antiviral discovery is advancing rapidly, yet many workflows remain poorly aligned with the biological complexity of respiratory RNA virus polymerases. To address this gap, we propose an RdRp-centered AI pipeline, structured as a staged framework that links target biology, molecular representation, structure-based prediction, benchmarking, and experimental validation. The goal is to move beyond docking scores toward experimentally actionable antiviral candidates. The integrated workflow is presented in Figure 4, whereas its stages, principal actions, and decision gates are detailed in Table 3 (Isert et al., 2023; Zhang et al., 2023; Harren et al., 2024; Sindt and Rognan, 2026; Tirosyan et al., 2026).

**FIGURE 4:**
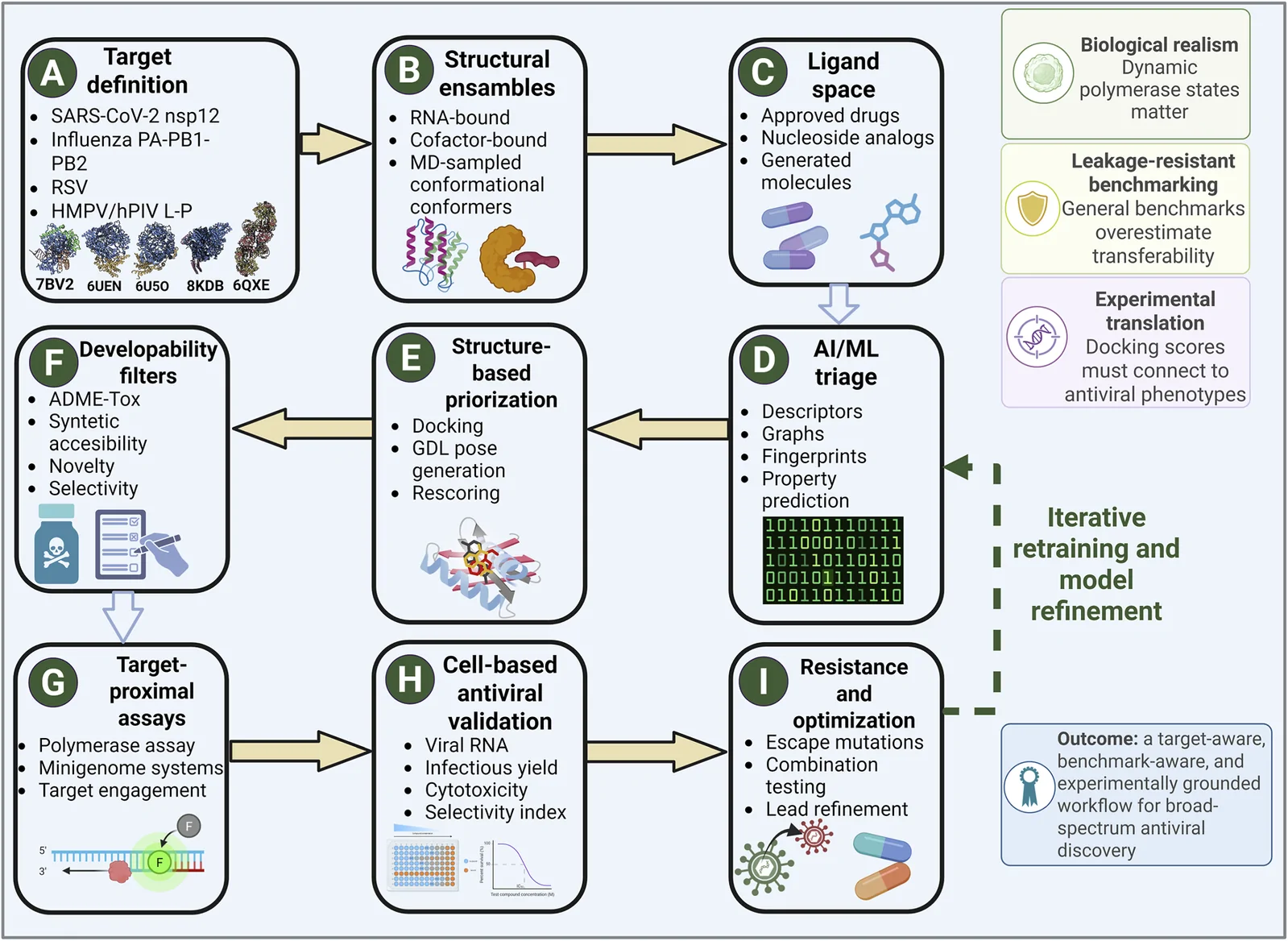
Proposed RdRp-centered AI pipeline for broad-spectrum respiratory antiviral discovery. The stage-gated workflow integrates nine connected components: **(A)** definition of relevant viral polymerase targets and mechanisms; **(B)** assembly of biologically relevant structures and conformational ensembles; **(C)** curation of approved drugs, nucleoside analogues, and generated molecules; **(D)** AI/ML-based triage using molecular descriptors, graphs, molecular fingerprints, and property prediction; **(E)** structure-based prioritization through docking, geometric deep learning, pose generation, and rescoring; **(F)** developability filtering based on ADME/Tox properties, synthetic accessibility, novelty, and selectivity; **(G)** target-proximal polymerase, minigenome, and target-engagement assays; **(H)** cell-based measurement of viral RNA, infectious yield, cytotoxicity, and selectivity index (SI = CC50/EC50); and **(I)** resistance profiling and candidate optimization. Experimental results are fed back into earlier stages through iterative retraining and model refinement. The framework emphasizes biological realism, leakage-resistant benchmarking, and experimental translation to produce target-aware and pharmacologically grounded antiviral candidates. Created with BioRender. Created in BioRender. De Jesus Gonzalez, L. A. (2026) https://BioRender.com/ve4nu1s.

**TABLE 3:** Stage-gated workflow that connects the computational components of the review to pharmacological validation.

Pipeline stage	Main action	Decision gate	Minimum stage-specific reporting requirement
Target definition	Specify viral family, polymerase complex, domain, receptor state, and inhibition hypothesis	Target must be biologically essential, mechanistically justified, and experimentally assayable	Report virus, target subunit or domain, cofactors, polymerase state, and the specific inhibition hypothesis, such as nucleotide-analog incorporation, chain termination, or allosteric blockade.
Structural ensemble	Compile apo/holo/RNA-bound/cofactor-bound/inhibitor-bound/MD conformations	At least one biologically relevant polymerase state must be represented, with uncertainty explicitly documented	Report PDB IDs or model sources, RNA presence, metal ions, cofactors, missing regions, protonation assumptions, and how ensemble conformations were selected.
Ligand-space curation	Approved drugs, nucleoside analogs, non-nucleoside inhibitors, fragments, natural products, generated molecules	Candidate forms must match the biological question and remain chemically tractable	Report compound source, stereochemistry, protonation and tautomer states, parent-drug/prodrug/active-metabolite form, and ADME/Tox or synthetic-accessibility filters.
ML triage	Apply drug-likeness, antiviral-likeness, ADME/Tox, and target-aware filters	Candidates should remain within the model applicability domain and pass uncertainty-based triage	Report training data, endpoint, split strategy, target-family coverage, applicability-domain definition, uncertainty estimates, and threshold used for progression.
Docking/GDL prediction	Run docking, pose-generation models, ensemble rescoring, and interaction analysis	Ranking must not rely on a single best score; pose plausibility and conserved mechanistic contacts must be assessed	Report software or model version, binding site, grid or search space, receptor state, ensemble members, controls, scoring functions, pose-selection criteria, and whether RNA/metals/cofactors were included.
Benchmark stress test	Use scaffold splits, target-family splits, external viral sets, and uncertainty estimates	Performance must be distinguished between interpolation and viral-target generalization	Report benchmark dataset, endpoint units, split type, leakage controls, external test set, evaluation metrics, confidence intervals when available, and failure cases.
Biochemical validation	Polymerase, capping, or minigenome assays	Target-proximal activity must be concentration-dependent and mechanistically interpretable	Report enzyme or replicase components, substrate/template design, nucleotide and metal conditions, positive control, concentration-response curve, IC50 or Ki, replicate number, and mechanism-specific readout.
Cellular validation	Viral RNA, infectious yield, antigen/reporter assays, cytotoxicity, selectivity index	Antiviral activity must occur at non-toxic concentrations and should be supported by infectious-virus or orthogonal readouts	Report virus strain or variant, cell model, MOI, treatment timing, assay endpoint, EC_50_, CC_50_ measured in parallel, selectivity index, positive control, and biological replicates.
Resistance profiling	Escape selection, mutant testing, conservation analysis	Resistance liability and fitness cost must be compatible with further optimization	Report mutation identity, selection or reverse-genetics method, sequencing confirmation, susceptibility fold-change, viral fitness, passage history, and cross-resistance when applicable.
Iterative optimization	Feed experimental results back into model and generative design loop	Only candidates meeting predefined biochemical, cellular, cytotoxicity, and resistance criteria should advance	Report the advance/stop criteria, experimental data used for retraining, model updates, structure-activity relationships, and whether new analogs improve potency, selectivity, or resistance profile.

RdRp-centered AI studies should report the receptor state, presence of RNA, cofactors and metals, ligand preparation, dataset composition, splitting strategy, applicability domain, docking or model-validation procedures, experimental validation level, cytotoxicity, and evidence supporting the proposed mechanism of action.

Detailed experimental standards are summarized in Table 2; this table provides the minimum stage-specific information needed to make the proposed pipeline reproducible and actionable.

The first stage is target definition. The pipeline begins by specifying the polymerase system, viral scope, and inhibition mechanism. For SARS-CoV-2, this may include nsp12-nsp7-nsp8, RNA, and relevant cofactors. For influenza, targets may include the PA endonuclease, the PB1 polymerase region, the PB2 cap-binding sites, or intersubunit interfaces. For RSV, hMPV, and HPIV, targets may include L protein domains, L-P interfaces, capping domains, or allosteric pockets (Isert et al., 2023; Zhang et al., 2023; Harren et al., 2024; Sindt and Rognan, 2026; Tirosyan et al., 2026).

The second stage is structural ensemble construction. Rather than selecting a single static receptor, the pipeline should compile biologically meaningful conformations: apo, holo, RNA-bound, nucleotide-bound, cofactor-bound, inhibitor-bound, and MD-derived states, where available. Structures should be annotated with resolution, missing regions, cofactors, metals, RNA, protonation, and pocket relevance (Isert et al., 2023; Zhang et al., 2023; Harren et al., 2024; Sindt and Rognan, 2026; Tirosyan et al., 2026).

The third stage is ligand-space definition. The candidate space should include approved drugs, investigational antivirals, nucleoside and nucleotide analogs, non-nucleoside inhibitors, capping-domain inhibitors, fragment libraries, natural products, and generated molecules. Each ligand should be standardized, protonated, tautomerized, and filtered for physicochemical and ADME properties (Isert et al., 2023; Zhang et al., 2023; Harren et al., 2024; Sindt and Rognan, 2026; Tirosyan et al., 2026).

The fourth stage is ML-enabled triage. Ligand libraries can be filtered using models for drug-likeness, ADME/Tox, antiviral-likeness, scaffold novelty, synthetic accessibility, and the presence of undesirable substructures. If target-specific data are available, supervised models can prioritize compounds that resemble known polymerase inhibitors. If data are sparse, transfer learning or similarity-informed models can be used, but predictions should include warnings about applicability domains (Isert et al., 2023; Zhang et al., 2023; Harren et al., 2024; Sindt and Rognan, 2026; Tirosyan et al., 2026).

The fifth stage is structure-based prediction. Docking, GDL pose-generation models, and ML rescoring can be applied across the structural ensemble. Compounds should not be ranked solely by their best score; they should be evaluated for pose plausibility, conserved contacts, robustness to receptor state, avoidance of artifact-prone interactions, and compatibility with the known polymerase mechanism (Isert et al., 2023; Zhang et al., 2023; Harren et al., 2024; Sindt and Rognan, 2026; Tirosyan et al., 2026).

The sixth stage is benchmark-aware model evaluation. Before using a model prospectively, its relevance to the antiviral task should be evaluated. This includes checking whether viral targets were present in training, whether similar ligands appear in the training and test sets, and whether performance is reported by target class. The seventh and eighth stages are experimental validation and iterative learning, in which biochemical assays, cell-based assays, resistance profiling, and model retraining form a design-make-test-analyze loop (Isert et al., 2023; Zhang et al., 2023; Harren et al., 2024; Sindt and Rognan, 2026; Tirosyan et al., 2026).

### 13.1 A composite prioritization score for antiviral candidates

The preceding analysis argues against ranking candidates by any single computational metric, yet a review that only criticizes RMSE or docking-based ranking offers limited practical guidance (Liu et al., 2017; Su et al., 2019; Harren et al., 2024). We therefore propose a Composite Antiviral Prioritization Score (CAPS) that operationalizes the pipeline’s decision gates as an explicit, transparent scheme. Each candidate is evaluated across six dimensions, each normalized to a common interval: (i) predicted binding and pose plausibility derived from docking or geometric deep learning; (ii) benchmark reliability, which penalizes models validated only on non-viral, leakage-prone datasets; (iii) biochemical target engagement, measured as polymerase inhibition; (iv) cellular antiviral activity in infected cells; (v) selectivity, computed from the CC_50_/EC_50_ ratio; and (vi) resistance-barrier robustness (Isert et al., 2023; Von Delft et al., 2023; Landrum and Riniker, 2024). At each decision stage, the applicable experimental dimensions function as hard gates rather than as compensable additive terms. A candidate that fails a predefined minimum requirement, such as measurable biochemical target engagement or an acceptable selectivity index, is classified as non-advancing regardless of its computational performance (Smee et al., 2017; Von Delft et al., 2023). Only candidates that pass the applicable gates receive a CAPS value, calculated as CAPS = Σwᵢsᵢ, where each dimension-specific score, sᵢ, is normalized to the interval [0,1], each weight, wᵢ, is non-negative, and Σwᵢ = 1. The combined weight assigned to biochemical, cellular, selectivity, and resistance evidence must exceed that assigned to computational binding and benchmark-reliability dimensions. All thresholds and weights must be specified *a priori* for each campaign and reported alongside the resulting rankings.

CAPS is intended as a decision framework rather than a validated numerical index, and the specific weights should be calibrated to the target, the available assays, and the stage of a given campaign. Its value is conceptual: it makes explicit that computational evidence prioritizes candidates but does not substitute for pharmacological validation, and it forces each stage of the pipeline to report a defined, minimally comparable output rather than an isolated affinity estimate.

### 13.2 Worked example: applying the RdRp-centered pipeline to SARS-CoV-2 nsp12

To make the proposed RdRp-centered pipeline more actionable, we illustrate its application using SARS-CoV-2 nsp12 as a worked example. This target was selected because several stages of the pipeline are already supported by experimentally determined structures, biochemical evidence, and resistance data (Gao et al., 2020; Yin et al., 2020; Kokic et al., 2021; Gandhi et al., 2022; Stevens et al., 2022). Published studies have implemented individual components of this workflow. For example, Rogers et al. (2023) performed ultra-large-scale docking against SARS-CoV-2 targets, including RdRp, and combined AutoDock-GPU scoring with RFScore v3 and DUD-E machine-learning rescoring (Rogers et al., 2023). Similarly, Van and Nguyen (2025) used a multi-model machine-learning framework to prioritize FDA-approved drugs against norovirus RdRp, followed by docking and molecular dynamics analysis (Van and Nguyen, 2025). However, these studies remain primarily computational and do not complete the full translational cascade from prediction to validation of biochemical, cellular, cytotoxicity, and resistance. The following example therefore integrates published evidence on RdRps with explicitly proposed validation steps.

In the target-definition stage, the pipeline would focus on the SARS-CoV-2 nsp12 catalytic core, including conserved motifs A–G, while also considering the NiRAN domain and the nsp7/nsp8 cofactors that stabilize the active polymerase complex (Gao et al., 2020; Hillen et al., 2020). The structural-ensemble stage would integrate complementary cryo-EM structures: the nsp12–nsp7–nsp8 complex without RNA, represented by PDB 6M71 (Gao et al., 2020); the active RNA-bound polymerase complex, represented by PDB 6YYT (Hillen et al., 2020); and the remdesivir-containing elongation complex, represented by PDB 7BV2 (Yin et al., 2020). Together, these structures allow candidate ligands to be evaluated not against a single rigid receptor, but against functionally distinct polymerase states relevant to nucleotide incorporation, RNA positioning, and inhibitor-induced stalling.

Ligand preparation should distinguish between pharmacological and mechanistic representations. Parent drugs or prodrugs should be evaluated for drug-likeness, exposure, and ADME/toxicity properties, whereas nucleotide analogs should be modeled in their intracellular active triphosphate forms when the goal is to interrogate polymerase recognition and incorporation. For example, remdesivir should be mechanistically linked to remdesivir triphosphate/RMP incorporation (Gordon et al., 2020; Yin et al., 2020; Kokic et al., 2021), whereas molnupiravir should be considered via NHC-triphosphate and its mutagenic pairing behavior (Kabinger et al., 2021). This distinction prevents conflating systemic pharmacokinetics with the chemical form that is recognized by the RdRp.

During the computational stage, ML triage would prioritize candidates within the applicability domain of the RdRp-relevant training data, while docking or geometric deep learning predictions would be used only as ranking tools rather than as evidence of antiviral activity. For nsp12, PDB 7BV2 should not be treated as a simple non-covalent redocking control because remdesivir is incorporated into the RNA product strand (Yin et al., 2020). Instead, it should serve as a structural benchmark for active-site geometry, RNA positioning, metal coordination, and consistency with the known mechanism of remdesivir (Yin et al., 2020; Kokic et al., 2021). Conventional pose-recovery benchmarks should be reserved for ligands with experimentally determined non-covalent binding modes. Candidate ranking would then be integrated into the composite scoring framework described above, which combines predicted affinity, pose stability, chemical tractability, uncertainty, and translational feasibility.

The first experimental gate would be a recombinant nsp12–nsp7–nsp8 primer-extension polymerase assay to confirm direct inhibition of RNA synthesis and, for nucleotide analogs, to define the mechanism of incorporation or delayed chain termination (Gordon et al., 2020; Lu et al., 2020). Remdesivir serves as a useful reference because biochemical and structural studies show that polymerase stalls after additional nucleotide incorporation rather than immediately terminating (Gordon et al., 2020; Kokic et al., 2021). The second gate would assess antiviral activity and selectivity in infected-cell models by measuring EC_50_ and CC_50_, ideally using both a permissive screening cell line and a respiratory-relevant model such as Calu-3 or primary airway epithelial cells (Pruijssers et al., 2020; Sheahan et al., 2020). The third gate would evaluate resistance by testing known or plausible nsp12 substitutions associated with altered remdesivir susceptibility, including S759A, V792I, and E802D, and by measuring viral fitness costs (Gandhi et al., 2022; Stevens et al., 2022). Only candidates that retain biochemical activity, cellular selectivity, and an acceptable resistance profile would proceed to iterative optimization.

This worked example clarifies which parts of the pipeline are already supported by published evidence for RdRps and which remain prospective. It also shows how the same framework could later be adapted to less-characterized respiratory polymerases, such as influenza PB1 or the RSV L protein, once comparable structural, biochemical, and resistance benchmarks become available.

## 14 Conclusions and future perspectives

The rapid advancement of AI-guided antiviral discovery depends on the alignment of computational models with the biological intricacies of viral targets. Polymerases of respiratory RNA viruses are ideally suited for assessing this transition. They are essential, conserved, mechanistically comprehensible, and pharmacologically accessible; nonetheless, they are also dynamic, cofactor-dependent, RNA-interacting, and inadequately represented in standard protein-ligand standards (Yang et al., 2022; Zhang et al., 2023; Bande and Baday, 2024; Harren et al., 2024; Tirosyan et al., 2026).

Future progress will depend on prospectively testing whether models trained and benchmarked under virologically realistic conditions enrich experimentally tractable candidates across distinct respiratory-virus polymerases. Such studies should compare performance across relevant conformational states, quantify uncertainty and applicability domains, and use predefined biochemical, cellular, pharmacological, and resistance endpoints to determine where computational prioritization adds measurable value (Yang et al., 2022; Zhang et al., 2023; Bande and Baday, 2024; Harren et al., 2024; Tirosyan et al., 2026).

Within this framework, AI is most valuable as an iterative decision-support system that learns from experimental outcomes, identifies sources of failure, and refines subsequent candidate selection. Integrating computational modeling with experimental virology and pharmacology may therefore accelerate RdRp-centered discovery and generate the evidence needed to advance robust, broad-spectrum antiviral candidates.

## References

[B1] Arús-Pous J. Patronov A. Bjerrum E. J. Tyrchan C. Reymond J.-L. Chen H. (2020). SMILES-Based deep generative scaffold decorator for *de-novo* drug design. J. Cheminform 12, 38. 10.1186/s13321-020-00441-8 PMC7260788 33431013

[B2] Azam S. S. Abbasi S. W. Batool M. (2014). Structure modeling and docking study of HCV NS5B-3a RNA polymerase for the identification of potent inhibitors. Med. Chem. Res. 23, 618–627. 10.1007/s00044-013-0666-5

[B3] Bagal V. Aggarwal R. Vinod P. K. Priyakumar U. D. (2022). MolGPT: molecular generation using a transformer-decoder model. J. Chem. Inf. Model. 62, 2064–2076. 10.1021/acs.jcim.1c00600 34694798

[B4] Bande A. Y. Baday S. (2024). Accelerating molecular docking using machine learning methods. Mol. Inf. 43, e202300167. 10.1002/minf.202300167 38850231

[B5] Beck B. R. Shin B. Choi Y. Park S. Kang K. (2020). Predicting commercially available antiviral drugs that May act on the novel coronavirus (SARS-CoV-2) through a drug-target interaction deep learning model. Comput. Struct. Biotechnol. J. 18, 784–790. 10.1016/j.csbj.2020.03.025 32280433 PMC7118541

[B6] Bhat A. R. Ahmed S. (2025). Artificial intelligence (AI) in drug design and discovery: a comprehensive review. Silico Res. Biomed. 1, 100049. 10.1016/j.insi.2025.100049

[B7] Bickerton G. R. Paolini G. V. Besnard J. Muresan S. Hopkins A. L. (2012). Quantifying the chemical beauty of drugs. Nat. Chem. 4, 90–98. 10.1038/nchem.1243 22270643 PMC3524573

[B8] Blaschke T. Arús-Pous J. Chen H. Margreitter C. Tyrchan C. Engkvist O. (2020). REINVENT 2.0: an AI Tool for *de novo* Drug Design. J. Chem. Inf. Model. 60, 5918–5922. 10.1021/acs.jcim.0c00915 33118816

[B9] Bongini P. Bianchini M. Scarselli F. (2021). Molecular generative graph neural networks for drug discovery. Neurocomputing 450, 242–252. 10.1016/j.neucom.2021.04.039

[B10] Bonneux B. Jacoby E. Ceconi M. Stobbelaar K. Delputte P. Herschke F. (2024). Direct-acting antivirals for RSV treatment, a review. Antivir. Res. 229, 105948. 10.1016/j.antiviral.2024.105948 38972604

[B11] Bonomini A. Mercorelli B. Loregian A. (2025). Antiviral strategies against influenza virus: an update on approved and innovative therapeutic approaches. Cell. Mol. Life Sci. 82, 75. 10.1007/s00018-025-05611-1 PMC11825441 39945883

[B12] Bouysset C. Fiorucci S. (2021). ProLIF: a library to encode molecular interactions as fingerprints. J. Cheminform 13, 72. 10.1186/s13321-021-00548-6 PMC8466659 34563256

[B13] Brown N. Fiscato M. Segler M. H. S. Vaucher A. C. (2019). GuacaMol: benchmarking Models for *de novo* Molecular Design. J. Chem. Inf. Model. 59, 1096–1108. 10.1021/acs.jcim.8b00839 30887799

[B14] Chakraborty C. Bhattacharya M. Lee S.-S. Wen Z.-H. Lo Y.-H. (2024). The changing scenario of drug discovery using AI to deep learning: recent advancement, success stories, collaborations, and challenges. Mol. Ther. - Nucleic Acids 35, 102295. 10.1016/j.omtn.2024.102295 PMC11386122 39257717

[B15] Chen E. A. Zhang Y. (2025). Can deep learning blind docking methods be used to predict allosteric compounds? J. Chem. Inf. Model. 65, 3737–3748. 10.1021/acs.jcim.5c00331 PMC12004537 40167386

[B16] Chen Y. Wang Z. Wang L. Wang J. Li P. Cao D. (2023). Deep generative model for drug design from protein target sequence. J. Cheminform 15, 38. 10.1186/s13321-023-00702-2 PMC10052801 36978179

[B17] Corso G. Stärk H. Jing B. Barzilay R. Jaakkola T. (2023). Diffdock: Diffusion Steps, Twists, and Turns for Molecular Docking. Proc. Mach. Learn. Res. 202, 6388–6410. 10.48550/arXiv.2210.01776

[B18] Cousins H. C. Nayar G. Altman R. B. (2024). Computational approaches to drug repurposing: methods, challenges, and opportunities. Annu. Rev. Biomed. Data Sci. 7, 15–29. 10.1146/annurev-biodatasci-110123-025333 38598857

[B19] De Jesús-González L. A. Santos-Mena A. O. Farfán-Morales C. N. León-Juárez M. Reyes-Ruiz J. M. Osuna-Ramos J. F. (2026). A network-driven antiviral discovery pipeline for emerging RNA viruses: a critical perspective from the Red Mexicana de Desarrollo de Antivirales (RMDA). Expert Opin. Drug Discov. 21 (3), 285–305. 10.1080/17460441.2026.2716137 42599129

[B20] Dong T. Yang Z. Zhou J. Chen C. Y.-C. (2023). Equivariant flexible modeling of the protein–ligand binding pose with geometric deep learning. J. Chem. Theory Comput. 19, 8446–8459. 10.1021/acs.jctc.3c00273 37938978

[B21] Fleming P. Ivanov A. A. (2026). Target discovery and drug design in the era of artificial intelligence. Med. Chem. Res. 37, 103245. 10.1007/s00044-026-03562-1 PMC13563285 42446537

[B22] Francoeur P. G. Masuda T. Sunseri J. Jia A. Iovanisci R. B. Snyder I. (2020). Three-dimensional convolutional neural networks and a cross-docked data set for structure-based drug design. J. Chem. Inf. Model. 60, 4200–4215. 10.1021/acs.jcim.0c00411 32865404 PMC8902699

[B23] Gandhi S. Klein J. Robertson A. J. Peña-Hernández M. A. Lin M. J. Roychoudhury P. (2022). *De novo* emergence of a remdesivir resistance mutation during treatment of persistent SARS-CoV-2 infection in an immunocompromised patient: a case report. Nat. Commun. 13, 1547. 10.1038/s41467-022-29104-y PMC8930970 35301314

[B24] Gao K. Nguyen D. D. Tu M. Wei G.-W. (2020). Generative network complex for the automated generation of drug-like molecules. J. Chem. Inf. Model. 60, 5682–5698. 10.1021/acs.jcim.0c00599 32686938 PMC8142330

[B25] Gaulton A. Bellis L. J. Bento A. P. Chambers J. Davies M. Hersey A. (2012). ChEMBL: a large-scale bioactivity database for drug discovery. Nucleic Acids Res. 40, D1100–D1107. 10.1093/nar/gkr777 21948594 PMC3245175

[B26] Gilson M. K. Liu T. Baitaluk M. Nicola G. Hwang L. Chong J. (2016). BindingDB in 2015: a public database for medicinal chemistry, computational chemistry and systems pharmacology. Nucleic Acids Res. 44, D1045–D1053. 10.1093/nar/gkv1072 26481362 PMC4702793

[B27] Gordon C. J. Tchesnokov E. P. Woolner E. Perry J. K. Feng J. Y. Porter D. P. (2020). Remdesivir is a direct-acting antiviral that inhibits RNA-Dependent RNA polymerase from severe acute respiratory syndrome coronavirus 2 with high potency. J. Biol. Chem. 295, 6785–6797. 10.1074/jbc.RA120.013679 32284326 PMC7242698

[B28] Graber D. Stockinger P. Meyer F. Mishra S. Horn C. Buller R. (2025). Resolving data bias improves generalization in binding affinity prediction. Nat. Mach. Intell. 7, 1713–1725. 10.1038/s42256-025-01124-5 PMC12552120 41143208

[B29] Gupta A. Müller A. T. Huisman B. J. H. Fuchs J. A. Schneider P. Schneider G. (2018). Generative Recurrent Networks for *de novo* Drug Design. Mol. Inf. 37, 1700111. 10.1002/minf.201700111 29095571 PMC5836943

[B30] Harren T. Gutermuth T. Grebner C. Hessler G. Rarey M. (2024). Modern machine‐learning for binding affinity estimation of protein–ligand complexes: progress, opportunities, and challenges. WIREs Comput. Mol. Sci. 14, e1716. 10.1002/wcms.1716

[B31] Hillen H. S. Kokic G. Farnung L. Dienemann C. Tegunov D. Cramer P. (2020). Structure of replicating SARS-CoV-2 polymerase. Nature 584, 154–156. 10.1038/s41586-020-2368-8 32438371

[B32] Huang D. Wang Y. Sun Y. Ji W. Zhang Q. Jiang Y. (2025a). A 4D tensor-enhanced multi-dimensional convolutional neural network for accurate prediction of protein-ligand binding affinity. Mol. Divers 29, 3041–3058. 10.1007/s11030-024-11044-y 39714563

[B33] Huang F. Xia Y. Wang Z. Yang L. Zhang W. (2025b). “Geometric heterogeneous graph neural network for protein-ligand binding affinity prediction,” in Proceedings of the 34th ACM International Conference on Information and Knowledge Management, Seoul, Republic of Korea, November 10-14, 2025, (ACM), 939–948. 10.1145/3746252.3761365

[B34] Irwin J. J. Tang K. G. Young J. Dandarchuluun C. Wong B. R. Khurelbaatar M. (2020). ZINC20—A free ultralarge-scale chemical database for ligand discovery. J. Chem. Inf. Model. 60, 6065–6073. 10.1021/acs.jcim.0c00675 33118813 PMC8284596

[B35] Isert C. Atz K. Schneider G. (2023). Structure-based drug design with geometric deep learning. Curr. Opin. Struct. Biol. 79, 102548. 10.1016/j.sbi.2023.102548 36842415

[B36] Izmailyan R. Matevosyan M. Khachatryan H. Shavina A. Gevorgyan S. Ghazaryan A. (2024). Discovery of new antiviral agents through artificial intelligence: *in vitro* and *in vivo* results. Antivir. Res. 222, 105818. 10.1016/j.antiviral.2024.105818 38280564

[B37] Jiang M. Zhang Z. Ma T. Hang H. Fan Y. Pang S. (2025). ProtGeoNet-Pocket: a binding site prediction approach integrating sequence, geometry, and graph structure. J. Chem. Inf. Model. 65, 10736–10753. 10.1021/acs.jcim.5c01568 40960030

[B38] Jo S. Signorile L. Kim S. Kim M.-S. Huertas O. Insa R. (2022). A study of drug repurposing to identify SARS-CoV-2 main protease (3CLpro) inhibitors. IJMS 23, 6468. 10.3390/ijms23126468 PMC9224295 35742913

[B39] Kabinger F. Stiller C. Schmitzová J. Dienemann C. Kokic G. Hillen H. S. (2021). Mechanism of molnupiravir-induced SARS-CoV-2 mutagenesis. Nat. Struct. Mol. Biol. 28, 740–746. 10.1038/s41594-021-00651-0 PMC8437801 34381216

[B40] Kc G. B. Bocci G. Verma S. Hassan M. M. Holmes J. Yang J. J. (2021). A machine learning platform to estimate anti-SARS-CoV-2 activities. Nat. Mach. Intell. 3, 527–535. 10.1038/s42256-021-00335-w

[B41] Kokic G. Hillen H. S. Tegunov D. Dienemann C. Seitz F. Schmitzova J. (2021). Mechanism of SARS-CoV-2 polymerase stalling by remdesivir. Nat. Commun. 12, 279. 10.1038/s41467-020-20542-0 PMC7804290 33436624

[B42] Krishnan A. Vinod D. (2024). Graph convolutional capsule regression (GCCR): a model for accelerated filtering of novel potential candidates for SARS-CoV-2 based on binding affinity. Curr. Comput. Aided Drug Des. 20, 33–41. 10.2174/1573409919666230331083953 37005531

[B43] Lam J. H. Katritch V. (2025). Navigating structure-based drug discovery with emerging innovations in physics- and knowledge-based approaches. Npj Drug Discov. 2, 29. 10.1038/s44386-025-00031-4 PMC12669043 41341471

[B44] Landrum G. A. Riniker S. (2024). Combining IC50 or *K I* values from different sources is a source of significant noise. J. Chem. Inf. Model. 64, 1560–1567. 10.1021/acs.jcim.4c00049 PMC10934815 38394344

[B45] Li Y. Pei J. Lai L. (2021). Structure-based *de novo* drug design using 3D deep generative models. Chem. Sci. 12, 13664–13675. 10.1039/D1SC04444C PMC8549794 34760151

[B46] Li Y. Li L. Wang S. Tang X. (2023). EQUIBIND: a geometric deep learning-based protein-ligand binding prediction method. DD&T 17, 363–364. 10.5582/ddt.2023.01063 37766553

[B47] Li G. Yuan Y. Zhang R. (2025). A spatial-temporal graph attention network for protein–ligand binding affinity prediction based on molecular geometry. Multimed. Syst. 31, 94. 10.1007/s00530-024-01650-z

[B48] Li J. Guan X. Zhang O. Sun K. Wang Y. Bagni D. (2026a). Leak proof PDBBind: a reorganized data set of protein–ligand complexes for more generalizable binding affinity prediction. J. Phys. Chem. B 130, 730–740. 10.1021/acs.jpcb.5c08598 PMC13430955 41486605

[B49] Li J. Jiang H. Ma W. Bi X. Chen R. Lu W. (2026b). Geometric deep learning for protein-ligand affinity prediction with hybrid message passing strategies. IEEE J. Biomed. Health Inf. 30, 5507–5519. 10.1109/JBHI.2025.3594210 40748800

[B50] Li J. Lu R. Tan Y. Liang P. Liu B. Gu S. (2026c). FlowDock: a unified flow-based framework for flexible protein-ligand docking and binding affinity prediction. Acta Pharm. Sin. B 16, 4067–4082. 10.1016/j.apsb.2026.04.009 PMC13366307 42453414

[B51] Liao Z. You R. Huang X. Yao X. Huang T. Zhu S. (2019). “DeepDock: enhancing ligand-protein interaction prediction by a combination of ligand and structure information,” in 2019 IEEE International Conference on Bioinformatics and Biomedicine (BIBM), San Diego, California, November 18-21, 2019. (San Diego, CA, USA: IEEE), 311–317. 10.1109/BIBM47256.2019.8983365

[B52] Lim H. Kim S. Lee S. (2025). “CheapNet: cross-Attention on hierarchical representations for efficient protein-ligand binding affinity prediction,” in The Thirteenth International Conference on Learning Representations (ICLR 2025), Singapore, April 24-28, 2025. Available online at: https://openreview.net/forum?id=A1HhtITVE (Accessed August 15, 2026).

[B53] Liu Y. Pan Z. (2025). Dual gated graph learning improves binding affinity estimation from three dimensional protein ligand complexes. J. Technol. Innovation Eng. 1, 100011. 10.63887/jtie.2025.1.8.11

[B54] Liu T. Lin Y. Wen X. Jorissen R. N. Gilson M. K. (2007). BindingDB: a web-accessible database of experimentally determined protein-ligand binding affinities. Nucleic Acids Res. 35, D198–D201. 10.1093/nar/gkl999 17145705 PMC1751547

[B55] Liu Z. Su M. Han L. Liu J. Yang Q. Li Y. (2017). Forging the basis for developing protein–ligand interaction scoring functions. Acc. Chem. Res. 50, 302–309. 10.1021/acs.accounts.6b00491 28182403

[B56] Liu W. Tian W. Lim T. S. Choong Y. S. (2026a). AAPPE: protein-ligand binding affinity prediction leveraging amino acid pair positional encoding in deep learning. J. Chem. Inf. Model 66, 867–880. 10.1021/acs.jcim.5c01872 41124588

[B57] Liu Y. Tang H. Niu T. Wang J. (2026b). A comparative study of deep learning and classical modeling approaches for protein-ligand binding pose and affinity prediction in coronavirus main proteases. J. Chem. Inf. Model 66, 731–743. 10.1021/acs.jcim.5c02481 PMC12801289 41429653

[B58] Lodh E. Majumder S. Chowdhury T. (2025). “CGDeepAff: deep learning-based approach for protein-ligand binding affinity estimation using CNN-GRU,” in 2025 8th International Conference on Electronics, Materials Engineering and Nano-Technology (Iementech), Kolkata, India, January 31–February 2, 2025. (Kolkata, India: IEEE), 1–6. 10.1109/IEMENTech65115.2025.10959578

[B59] Lodh E. Majumder S. Chowdhury T. De M. (2026). RLBindDeep: a ResNet-LSTM based novel framework for protein-ligand binding affinity prediction. J. Mol. Graph Model 144, 109282. 10.1016/j.jmgm.2026.109282 41534323

[B60] Loeffler H. H. He J. Tibo A. Janet J. P. Voronov A. Mervin L. H. (2024). Reinvent 4: modern AI–driven generative molecule design. J. Cheminform 16, 20. 10.1186/s13321-024-00812-5 38383444 PMC10882833

[B61] Lu G. Zhang X. Zheng W. Sun J. Hua L. Xu L. (2020). Development of a simple *in vitro* assay to identify and evaluate nucleotide analogs against SARS-CoV-2 RNA-dependent RNA polymerase. Antimicrob. Agents Chemother. 65. e01508-20. 10.1128/AAC.01508-20 33122171 PMC7927875

[B62] Lu L. Qin J. Chen J. Yu N. Miyano S. Deng Z. (2022). Recent computational drug repositioning strategies against SARS-CoV-2. Comput. Struct. Biotechnol. J. 20, 5713–5728. 10.1016/j.csbj.2022.10.017 36277237 PMC9575573

[B63] Lu W. Zhang J. Huang W. Zhang Z. Jia X. Wang Z. (2024). DynamicBind: predicting ligand-specific protein-ligand complex structure with a deep equivariant generative model. Nat. Commun. 15, 1071. 10.1038/s41467-024-45461-2 PMC10844226 38316797

[B64] Mahdizadeh S. J. Eriksson L. A. (2025). iScore: a ML-Based Scoring Function for *de novo* Drug Discovery. J. Chem. Inf. Model 65, 2759–2772. 10.1021/acs.jcim.4c02192 40036330 PMC11938276

[B65] Malone B. Urakova N. Snijder E. J. Campbell E. A. (2022). Structures and functions of coronavirus replication–transcription complexes and their relevance for SARS-CoV-2 drug design. Nat. Rev. Mol. Cell Biol. 23, 21–39. 10.1038/s41580-021-00432-z PMC8613731 34824452

[B66] McNutt A. T. Francoeur P. Aggarwal R. Masuda T. Meli R. Ragoza M. (2021). GNINA 1.0: molecular docking with deep learning. J. Cheminform 13, 43. 10.1186/s13321-021-00522-2 34108002 PMC8191141

[B67] Mohamed Abdul Cader J. Newton M. A. H. Rahman J. Mohamed Abdul Cader A. J. Sattar A. (2024). Ensembling methods for protein-ligand binding affinity prediction. Sci. Rep. 14, 24447. 10.1038/s41598-024-72784-3 39424851 PMC11489440

[B68] Molina D. M. Jafari R. Ignatushchenko M. Seki T. Larsson E. A. Dan C. (2013). Monitoring drug target engagement in cells and tissues using the cellular thermal shift assay. Science 341, 84–87. 10.1126/science.1233606 23828940

[B69] Morehead A. Liu J. Neupane P. Cheng J. (2026). Artificial intelligence methods for protein structure and interaction prediction: recent advances and challenges. Curr. Opin. Struct. Biol. 98, 103247. 10.1016/j.sbi.2026.103247 41844067 PMC13002126

[B70] Murgueitio M. S. Bermudez M. Mortier J. Wolber G. (2012). *In silico* virtual screening approaches for anti-viral drug discovery. Drug Discov. Today Technol. 9, e219–e225. 10.1016/j.ddtec.2012.07.009 24990575 PMC7105918

[B71] Mysinger M. M. Carchia M. Irwin J. J. Shoichet B. K. (2012). Directory of useful decoys, enhanced (DUD-E): better ligands and decoys for better benchmarking. J. Med. Chem. 55, 6582–6594. 10.1021/jm300687e 22716043 PMC3405771

[B72] Olivecrona M. Blaschke T. Engkvist O. Chen H. (2017). Molecular *de-novo* design through deep reinforcement learning. J. Cheminform 9, 48. 10.1186/s13321-017-0235-x 29086083 PMC5583141

[B73] Ontiveros-Zúñiga L. F. Sosa-Mondragon S. I. Osuna-Ramos J. F. Llanas-Vázquez R. S. Cañedo-Figueroa D. M. Velázquez-Cervantes M. A. (2026). Therapeutic targeting of respiratory syncytial virus: current pharmacological options and future directions in antiviral development. Microbiol. Res. 309, 128536. 10.1016/j.micres.2026.128536 42068597

[B74] Ozdemir E. S. Jang H. Keskin O. Gursoy A. Nussinov R. (2026). Deep generative molecular design and its value in modern drug discovery. Expert Opin. Drug Discov. 21, 273–287. 10.1080/17460441.2026.2636192 41778634

[B75] Patel L. Shukla T. Huang X. Ussery D. W. Wang S. (2020). Machine learning methods in drug discovery. Molecules 25, 5277. 10.3390/molecules25225277 33198233 PMC7696134

[B76] Pathania S. Rawal R. K. Singh P. K. (2022). RdRp (RNA-Dependent RNA polymerase): a key target providing anti-virals for the management of various viral diseases. J. Mol. Struct. 1250, 131756. 10.1016/j.molstruc.2021.131756 34690363 PMC8520695

[B77] Peele K. A. Potla Durthi C. Srihansa T. Krupanidhi S. Ayyagari V. S. Babu D. J. (2020). Molecular docking and dynamic simulations for antiviral compounds against SARS-CoV-2: a computational study. Inf. Med. Unlocked 19, 100345. 10.1016/j.imu.2020.100345 32395606 PMC7211761

[B78] Popova M. Isayev O. Tropsha A. (2018). Deep reinforcement learning for *de novo* drug design. Sci. Adv. 4, eaap7885. 10.1126/sciadv.aap7885 30050984 PMC6059760

[B79] Praditya D. F. Waluyo D. Nozaki T. (2025). Reporter-expressing viruses for antiviral drug discovery research. Front. Cell. Infect. Microbiol. 15, 1645104. 10.3389/fcimb.2025.1645104 41479550 PMC12753968

[B80] Pruijssers A. J. George A. S. Schäfer A. Leist S. R. Gralinksi L. E. Dinnon K. H. (2020). Remdesivir inhibits SARS-CoV-2 in human lung cells and chimeric SARS-CoV expressing the SARS-CoV-2 RNA polymerase in mice. Cell Rep. 32, 107940. 10.1016/j.celrep.2020.107940 32668216 PMC7340027

[B81] Rahman J. Newton M. A. H. Ali M. E. Sattar A. (2024). Distance plus attention for binding affinity prediction. J. Cheminform 16, 52. 10.1186/s13321-024-00844-x 38735985 PMC11089753

[B82] Rahman J. Newton M. A. H. Mohamed Abdul Cader J. Islam M. K. B. Ali M. E. Sattar A. (2026). Harnessing angular geometry in deep learning for protein-ligand binding affinity prediction. Comput. Methods Programs Biomed. 278, 109282. 10.1016/j.cmpb.2026.109282 41691876

[B83] Ranganath A. Kim H. Shim H. Allen J. E. (2026). SLAB: simultaneous labeling and binding affinity prediction for protein–ligand structures. Digit. Discov. 5, 375–383. 10.1039/D5DD00248F

[B84] Rayka M. Latifi A. M. Mirzaei M. (2025). Assisting InSilicoDrug discovery through protein-ligand binding affinity prediction by convolutional neural networks. J. Apple Biotechnol. Rep. 12, 105–118. 10.30491/jabr.2023.405609.1651

[B85] Ren F. Ding X. Zheng M. Korzinkin M. Cai X. Zhu W. (2023). AlphaFold accelerates artificial intelligence powered drug discovery: efficient discovery of a novel CDK20 small molecule inhibitor. Chem. Sci. 14, 1443–1452. 10.1039/D2SC05709C PMC9906638 36794205

[B86] Rogers D. M. Agarwal R. Vermaas J. V. Smith M. D. Rajeshwar R. T. Cooper C. (2023). SARS-CoV2 billion-compound docking. Sci. Data 10, 173. 10.1038/s41597-023-01984-9 36977690 PMC10044124

[B87] Roney M. Mohd Aluwi M. F. F. (2024). The importance of *in-silico* studies in drug discovery. Intell. Pharm. 2, 578–579. 10.1016/j.ipha.2024.01.010

[B88] Sadybekov A. V. Katritch V. (2023). Computational approaches streamlining drug discovery. Nature 616, 673–685. 10.1038/s41586-023-05905-z 37100941

[B89] Savitski M. M. Reinhard F. B. M. Franken H. Werner T. Savitski M. F. Eberhard D. (2014). Tracking cancer drugs in living cells by thermal profiling of the proteome. Science 346, 1255784. 10.1126/science.1255784 25278616

[B90] Schenone M. Dančík V. Wagner B. K. Clemons P. A. (2013). Target identification and mechanism of action in chemical biology and drug discovery. Nat. Chem. Biol. 9, 232–240. 10.1038/nchembio.1199 23508189 PMC5543995

[B91] Schneuing A. Harris C. Du Y. Didi K. Jamasb A. Igashov I. (2024). Structure-based drug design with equivariant diffusion models. Nat. Comput. Sci. 4, 899–909. 10.1038/s43588-024-00737-x 39653846 PMC11659159

[B92] Sheahan T. P. Sims A. C. Zhou S. Graham R. L. Pruijssers A. J. Agostini M. L. (2020). An orally bioavailable broad-spectrum antiviral inhibits SARS-CoV-2 in human airway epithelial cell cultures and multiple coronaviruses in mice. Sci. Transl. Med. 12, eabb5883. 10.1126/scitranslmed.abb5883 32253226 PMC7164393

[B93] Sim J. Lee J. (2026). BA-Pred and RMSD-Pred: integrated graph neural network models for accurate protein-ligand binding affinity and binding pose prediction. J. Chem. Inf. Model 66, 3480–3495. 10.1021/acs.jcim.5c02591 41921096 PMC13080981

[B94] Sim J. Kim D. Kim B. Choi J. Lee J. (2025). Recent advances in AI-driven protein-ligand interaction predictions. Curr. Opin. Struct. Biol. 92, 103020. 10.1016/j.sbi.2025.103020 39999605

[B95] Sindt F. Rognan D. (2026). Structure-based virtual screening of ultra-large chemical spaces: advances and pitfalls. Eur. J. Med. Chem. 305, 118576. 10.1016/j.ejmech.2026.118576 41539275

[B96] Skalic M. Sabbadin D. Sattarov B. Sciabola S. De Fabritiis G. (2019). From target to drug: generative modeling for the multimodal structure-based ligand design. Mol. Pharm. 16, 4282–4291. 10.1021/acs.molpharmaceut.9b00634 31437001

[B97] Smee D. F. Hurst B. L. Evans W. J. Clyde N. Wright S. Peterson C. (2017). Evaluation of cell viability dyes in antiviral assays with RNA viruses that exhibit different cytopathogenic properties. J. Virol. Methods 246, 51–57. 10.1016/j.jviromet.2017.03.012 28359770 PMC5479350

[B98] Song G. Deng M. Chen Y. Jia S. Nie Z. (2025). NciaNet: a non-covalent interaction-aware graph neural network for the prediction of protein-ligand interaction in drug discovery. IEEE J. Biomed. Health Inf. 29, 6055–6068. 10.1109/JBHI.2025.3547741 40036511

[B99] Ståhl N. Falkman G. Karlsson A. Mathiason G. Boström J. (2019). Deep Reinforcement Learning for Multiparameter Optimization in *de novo* Drug Design. J. Chem. Inf. Model. 59, 3166–3176. 10.1021/acs.jcim.9b00325 31273995

[B100] Stärk H. Ganea O. Pattanaik L. Barzilay R. Jaakkola T. (2022). “EquiBind: geometric deep learning for drug binding structure prediction,” in Proceedings of the 39th International Conference on Machine Learning, Baltimore, MD, July 17–23, 2022. Editors Chaudhuri K. Jegelka S. Song L. Szepesvari C. Niu G. Sabato S. , 20503–20521. Available online at: https://proceedings.mlr.press/v162/stark22b.html (Accessed August 15, 2026).

[B101] Sterling T. Irwin J. J. (2015). ZINC 15 – ligand discovery for everyone. J. Chem. Inf. Model 55, 2324–2337. 10.1021/acs.jcim.5b00559 26479676 PMC4658288

[B102] Stevens L. J. Pruijssers A. J. Lee H. W. Gordon C. J. Tchesnokov E. P. Gribble J. (2022). Mutations in the SARS-CoV-2 RNA-Dependent RNA polymerase confer resistance to remdesivir by distinct mechanisms. Sci. Transl. Med. 14, eabo0718. 10.1126/scitranslmed.abo0718 35482820 PMC9097878

[B103] Su M. Yang Q. Du Y. Feng G. Liu Z. Li Y. (2019). Comparative assessment of scoring functions: the CASF-2016 update. J. Chem. Inf. Model. 59, 895–913. 10.1021/acs.jcim.8b00545 30481020

[B104] Sun J. Sun D. Yang Q. Wang D. Peng J. Guo H. (2025). A novel, covalent broad-spectrum inhibitor targeting human coronavirus mpro. Nat. Commun. 16, 4546. 10.1038/s41467-025-59870-4 PMC12081877 40374668

[B105] Ta L. Gosa L. Nathanson D. A. (2019). “Biosafety and biohazards: understanding biosafety levels and meeting safety requirements of a biobank,” in Biobanking. Editor Yong W. H. (New York, NY: Springer New York), 213–225. 10.1007/978-1-4939-8935-5_19 PMC7120677 30539447

[B106] Tanimoto S. Itoh S. G. Okumura H. (2022). State-of-the-Art molecular dynamics simulation studies of RNA-dependent RNA polymerase of SARS-CoV-2. IJMS 23, 10358. 10.3390/ijms231810358 36142270 PMC9499461

[B107] Te Velthuis A. J. W. (2014). Common and unique features of viral RNA-Dependent polymerases. Cell. Mol. Life Sci. 71, 4403–4420. 10.1007/s00018-014-1695-z 25080879 PMC4207942

[B108] Te Velthuis A. J. W. Fodor E. (2016). Influenza virus RNA polymerase: insights into the mechanisms of viral RNA synthesis. Nat. Rev. Microbiol. 14, 479–493. 10.1038/nrmicro.2016.87 27396566 PMC4966622

[B109] Tirosyan I. Gabrielyan Y. Petrosyan V. Vignuzzi M. Zakaryan H. (2026). Can artificial intelligence transform antiviral drug discovery? Drug Discov. Today 31, 104648. 10.1016/j.drudis.2026.104648 41875943

[B110] Tondar A. Sánchez-Herrero S. Bepari A. K. Bahmani A. Calvet Liñán L. Hervás-Marín D. (2024). Virtual screening of small molecules targeting BCL2 with machine learning, molecular docking, and MD simulation. Biomolecules 14, 544. 10.3390/biom14050544 38785951 PMC11118195

[B111] Trott O. Olson A. J. (2010). AutoDock vina: improving the speed and accuracy of docking with a new scoring function, efficient optimization, and multithreading. J. Comput. Chem. 31, 455–461. 10.1002/jcc.21334 19499576 PMC3041641

[B112] Van N. T. H. Nguyen M. T. (2025). A multi-model machine learning framework for identifying raloxifene as a novel RNA polymerase inhibitor from FDA-approved drugs. CIMB 47, 315. 10.3390/cimb47050315 PMC12110393 40699714

[B113] Verdonk M. L. Cole J. C. Hartshorn M. J. Murray C. W. Taylor R. D. (2003). Improved protein–ligand docking using GOLD. Proteins 52, 609–623. 10.1002/prot.10465 12910460

[B114] Vicenti I. Zazzi M. Saladini F. (2021). SARS-CoV-2 RNA-Dependent RNA polymerase as a therapeutic target for COVID-19. Expert Opin. Ther. Pat. 31, 325–337. 10.1080/13543776.2021.1880568 33475441 PMC7938656

[B115] Von Delft A. Hall M. D. Kwong A. D. Purcell L. A. Saikatendu K. S. Schmitz U. (2023). Accelerating antiviral drug discovery: lessons from COVID-19. Nat. Rev. Drug Discov. 22, 585–603. 10.1038/s41573-023-00692-8 37173515 PMC10176316

[B116] Wang G. Zhang H. Shao M. Feng Y. Cao C. Hu X. (2024). DeepTGIN: a novel hybrid multimodal approach using transformers and graph isomorphism networks for protein-ligand binding affinity prediction. J. Cheminform 16, 147. 10.1186/s13321-024-00938-6 39734235 PMC11684089

[B117] Wei L. Fu N. Song Y. Wang Q. Hu J. (2023). Probabilistic generative transformer language models for generative design of molecules. J. Cheminform 15, 88. 10.1186/s13321-023-00759-z 37749655 PMC10518939

[B118] Wong F. Krishnan A. Zheng E. J. Stärk H. Manson A. L. Earl A. M. (2022). Benchmarking alphafold‐enabled molecular docking predictions for antibiotic discovery. Mol. Syst. Biol. 18, MSB202211081. 10.15252/msb.202211081 PMC9446081 36065847

[B119] Xu X. Chen Y. Lu X. Zhang W. Fang W. Yuan L. (2022). An update on inhibitors targeting RNA-Dependent RNA polymerase for COVID-19 treatment: promises and challenges. Biochem. Pharmacol. 205, 115279. 10.1016/j.bcp.2022.115279 36209840 PMC9535928

[B120] Yang C. Chen E. A. Zhang Y. (2022). Protein–ligand docking in the machine-learning era. Molecules 27, 4568. 10.3390/molecules27144568 PMC9323102 35889440

[B121] Yin W. Mao C. Luan X. Shen D.-D. Shen Q. Su H. (2020). Structural basis for inhibition of the RNA-Dependent RNA polymerase from SARS-CoV-2 by remdesivir. Science 368, 1499–1504. 10.1126/science.abc1560 32358203 PMC7199908

[B122] Yoon J. Kim Y. Lee H. (2026). TUNA: a target-aware unified network for protein-ligand binding affinity prediction *via* multi-modal feature integration. IEEE J. Biomed. Health Inf. 30, 6499–6512. 10.1109/JBHI.2025.3643854 41385420

[B123] Zhang X. Zhang O. Shen C. Qu W. Chen S. Cao H. (2023). Efficient and accurate large library ligand docking with KarmaDock. Nat. Comput. Sci. 3, 789–804. 10.1038/s43588-023-00511-5 38177786

[B124] Zhang H. Ge C. Fisher D. Hien N. T. T. Musabaev E. Pronyuk K. (2025). Antiviral treatment for viral pneumonia: current drugs and natural compounds. Virology J. 22, 62. 10.1186/s12985-025-02666-1 PMC11887211 40050867

[B125] Zhou G. Gao Z. Ding Q. Zheng H. Xu H. Wei Z. (2023). Uni-Mol: A Universal 3D Molecular Representation Learning Framework. ChemRxiv. 10.26434/chemrxiv-2022-jjm0j-v4

[B126] Zhu W. Chen C. Z. Gorshkov K. Xu M. Lo D. C. Zheng W. (2020). RNA-dependent RNA polymerase as a target for COVID-19 drug discovery. SLAS Discov. 25, 1141–1151. 10.1177/2472555220942123 32660307 PMC7684788

